# Aluminum Distribution in Ferrierite Zeolites Influences the Performance of Methane Oxidation

**DOI:** 10.1002/anie.202506023

**Published:** 2025-06-25

**Authors:** Peipei Xiao, Xiaomin Tang, Hiroto Toyoda, Yilin Wang, Anmin Zheng, Lizhuo Wang, Jun Huang, Masato Sawada, Kengo Nakamura, Yong Wang, Hermann Gies, Toshiyuki Yokoi

**Affiliations:** ^1^ Institute of Integrated Research Institute of Science Tokyo 4259 Nagatsuta, Midori‐ku Yokohama 226‐8501 Japan; ^2^ State Key Laboratory of Magnetic Resonance Spectroscopy and Imaging, National Center for Magnetic Resonance in Wuhan, Innovation Academy for Precision Measurement Science and Technology Chinese Academy of Sciences Wuhan 430071 China; ^3^ Interdisciplinary Institute of NMR and Molecular Sciences, Hubei Province for Coal Conversion and New Carbon Materials, School of Chemistry and Chemical Engineering Wuhan University of Science and Technology Wuhan 430081 China; ^4^ School of Chemical and Biomolecular Engineering the University of Sydney Sydney New South Wales 2006 Australia; ^5^ iPEACE223 Inc. Konwa Building, 1‐12‐22 Tsukiji Chuo‐ku Tokyo 104‐0045 Japan

**Keywords:** Al distribution, Aluminosilicate zeolite, Ferrierite zeolite, Methane oxidation, Methanol‐to‐olefins

## Abstract

Transition‐metal‐free aluminosilicate FER‐type zeolite has been demonstrated to effectively catalyze methane to methanol using N_2_O as the oxidant with distorted tetra‐coordinated aluminum (Al_IV‐2_) and penta‐coordinated aluminum (Al_V_) as potential active sites. However, the specific effects of Al distribution on the active Al species have not been thoroughly investigated. Herein, aluminosilicate FER‐type zeolites with controllable Al distribution were developed. Al distribution, including the arrangement and location of Al atoms, was characterized using ^27^Al MQMAS/MAS and ^29^Si MAS NMR spectra. The arrangement of aluminum, particularly the isolated Al and paired Al in as‐synthesized samples, influenced the proximity between oxidative and acidic sites in H‐type samples. Al locations involved the specific positioning of bifunctional sites and affected the final product. The increased CH_4_ conversion at 250–275 °C of FER zeolite with Al preferential population at T4 sites confirmed the higher activity of Al species from T4 sites. Additionally, a higher proportion of Al atoms in 10‐ring channels facilitated the tandem conversion of methane to methanol on oxidative sites, followed by methanol to hydrocarbons on acidic sites at 300–375 °C. This study corroborated and expanded upon our recent research and highlighted the significant impact of Al distribution in FER zeolite on methane oxidation.

## Graphical abstract

FER‐type aluminosilicate zeolites with preferential Al occupancy at T4 site favor direct methane‐to‐methanol oxidation, while those with Al concentrated at T3 sites within 10‐ring channels facilitate tandem conversion of methane‐to‐methanol on oxidative sites, followed by methanol‐to‐hydrocarbons on acidic sites.

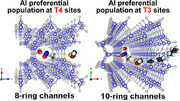

## Introduction

Zeolites are crystalline microporous materials composed of SiO_4_ and AlO_4_ tetrahedral (T) units arranged in a network of uniformly shaped micropores with adjustable topology and composition.^[^ ^1^, ^2^, ^3^ ^]^ Acid sites in zeolites are regularly generated when protons compensate for the negatively charged framework tetrahedrally coordinated Al atom (AlO_4_^−^).^[^ ^4^ ^]^ The Al distribution of zeolite has garnered significant attention as an important factor in influencing the activity and lifetime in acid catalysis,^[^ ^5^, ^6^ ^]^ redox reactions,^[^ ^7^, ^8^, ^9^ ^]^ and bifunctional catalysis.^[^ ^6^, ^10^, ^11^, ^12^, ^13^, ^14^, ^15^, ^16^, ^17^ ^]^ The concentration, location, and arrangement of Al atoms in a given structure of zeolite significantly affected the acid amount and local environment of acid sites.^[^ ^2^, ^17^, ^18^ ^]^ Accordingly, the activity and lifetime in certain reactions were profoundly impacted.^[^ ^19^ ^]^ Gounder and coworkers have found the effects of relative ratios of organic (*N*,*N*,*N*‐trimethyl‐1‐admantylammonium) and inorganic (Na^+^) cations on the arrangement of isolated and paired Al in CHA zeolite.^[^ ^20^ ^]^ Our previous work has reported that Al was preferentially located in narrow straight and/or sinusoidal channels in ZSM‐5 zeolite adjusted by various alcohols resulted in an extended lifetime in the MTO reaction.^[^ ^21^ ^]^ In addition, aside from the framework Al, the Al distribution included framework‐associated aluminum, distorted tetrahedral Al, and extra‐framework aluminum (EFAl), which encompasses partially uncoordinated Al (tri‐ and penta‐coordinated) and hexa‐coordinated Al lacking any bonds with framework oxygens.^[^ ^17^, ^22^ ^]^ In the case of metal‐loaded zeolites, the Al distribution of zeolite influenced the activity and selectivity in redox reactions, such as direct oxidation of methane to methanol (DMTM), partial oxidation of methane to syngas (POM), and selective catalytic reduction of NO_x_ with NH_3_ (SCR‐NH_3_).^[^ ^23^ ^]^ Zeolites with ordered microporous systems, various dimensions, superior (hydro)thermal stability, and adjustable anchor points are the ideal support for hosting diverse metal species, contributing to outstanding activity, unique shape selectivity, and tough stability and recyclability.^[^ ^23^ ^]^ Aluminosilicate zeolites are the preferred supports to exchange with metal cations because the Al content and its distribution can regulate the number and location of exchanged metal species, thus influencing the reactivity.^[^ ^23^ ^]^ In our previous work, dicopper and isolated Cu [Cu(OH)]^+^ species were adjusted by Al pairs‐rich and Al single‐rich AEI‐type zeolites; thus, different activity and selectivity were obtained in the methane oxidation reaction.^[^ ^24^, ^25^ ^]^ Besides, the location and size of the Rh species were determined by using ZSM‐5 zeolites prepared in the presence or absence of Na.^[^ ^21^ ^]^ The framework Al atoms were predominantly situated within the channel intersections, leading to relatively large Rh species and, consequently, a higher catalytic activity for the POM reaction.^[^ ^26^ ^]^ Furthermore, the metal species loaded on the zeolite via framework Al as an anchor can function as a bifunctional zeolite catalyst. In this context, the positioning of framework Al also plays a crucial role in both metal loading and the availability of acid sites. In our previous work, the spatial distance between Cu species and acid sites has been proved to directly influence the subsequent conversion of methanol to olefin.^[^ ^10^ ^]^ Additionally, the one‐pot synthesized Fe‐CHA zeolite without Na cations exhibited improved reaction performance in the direct oxidation of methane to olefins via methanol as an intermediate, compared to the zeolite synthesized with Na cations. This enhancement was attributed to the presence of distant Fe species and isolated acid sites in the tandem reaction.^[^ ^11^ ^]^

FER zeolite features a 2D pore system comprising a 10‐ring (10 R) pore (4.2 Å × 5.4 Å) along the [001] direction, which is intersected by an 8 R (3.5 Å × 4.8 Å) along the [010] direction.^[^ ^27^ ^]^ The FER cage is situated between two adjacent 10 R pores and is accessible solely through the 8 R window.^[^ ^28^ ^]^ FER zeolite exhibits exceptional thermal and chemical stability.^[^ ^29^ ^]^ The regulation and analysis of Al distribution in FER zeolite have been extensively researched.^[^ ^30^, ^31^, ^32^ ^]^ The adjustment of Al in FER zeolite can be achieved using different organic structure directing agents (OSDAs),^[^ ^2^, ^17^, ^30^, ^31^, ^33^ ^]^ introducing pore‐filling agents (PFA),^[^ ^28^ ^]^ adopting seeds^[^ ^34^ ^]^ and so on. There are four inequivalent T sites in a unit cell for FER zeolite.^[^ ^35^ ^] 27^Al MQMAS/MAS NMR spectra and the thermodynamic stability assessments based on density functional theory (DFT) calculations can be employed to confirm the location of framework Al for FER zeolite.^[^ ^28^, ^31^ ^]^ In our latest research, we reported that transition‐metal‐free aluminosilicate FER‐type zeolite effectively and stably catalyzed methane to methanol with minimal tandem reaction converting methanol to olefins.^[^ ^29^ ^]^ We attributed the limited occurrence of tandem reaction to the 2D topological structure of FER zeolite and the Al distribution of commercial FER zeolite from Zeolyst (CP914C).

In this work, FER zeolites with different Al distributions were synthesized using dioxane^[^ ^28^ ^]^ and pyrrolidine^[^ ^36^ ^]^ as PFA and OSDA, respectively. The Al distribution was evaluated by ^27^Al MQMAS/MAS NMR spectra, ^29^Si MAS NMR spectra, and DFT calculations. The location of framework Al was demonstrated to influence the formation of active Al species on the extra framework, resulting in different activity in methane oxidation to methanol. Moreover, the location and arrangement of Al atoms within the FER zeolite were studied for the effects on the subsequent reactions at appropriate temperatures.

## Results and Discussion

### Catalysts Characterization

**Figure 1:**
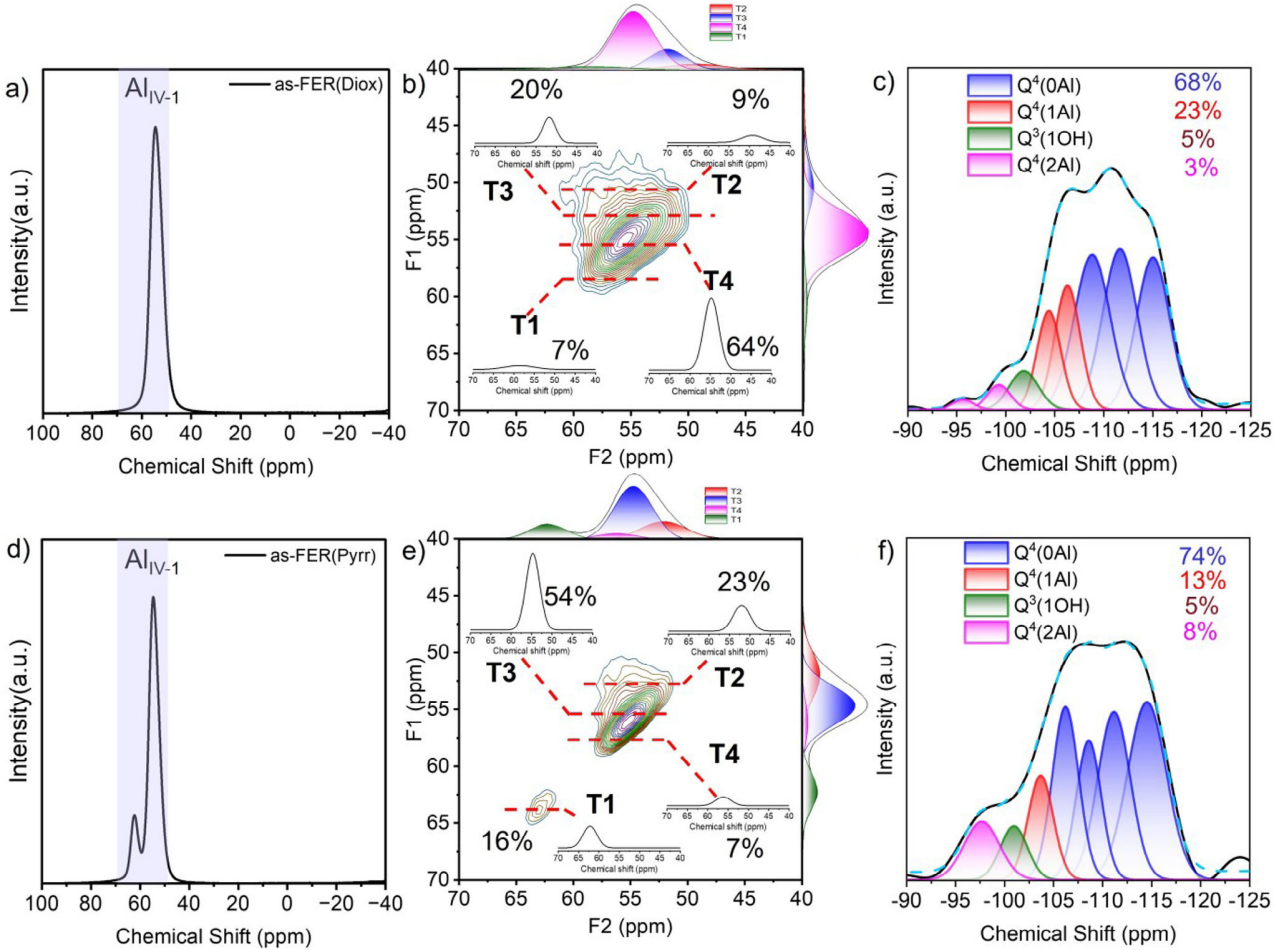
a) ^27^Al MAS NMR spectrum, b) ^27^Al MQMAS NMR spectrum, and c) deconvolution of ^29^Si MAS NMR spectrum of as‐FER(Diox). d) ^27^Al MAS NMR spectrum, e) ^27^Al MQMAS NMR spectrum, and f) deconvolution of ^29^Si MAS NMR spectrum of as‐FER(Pyrr).

**Scheme 1:**
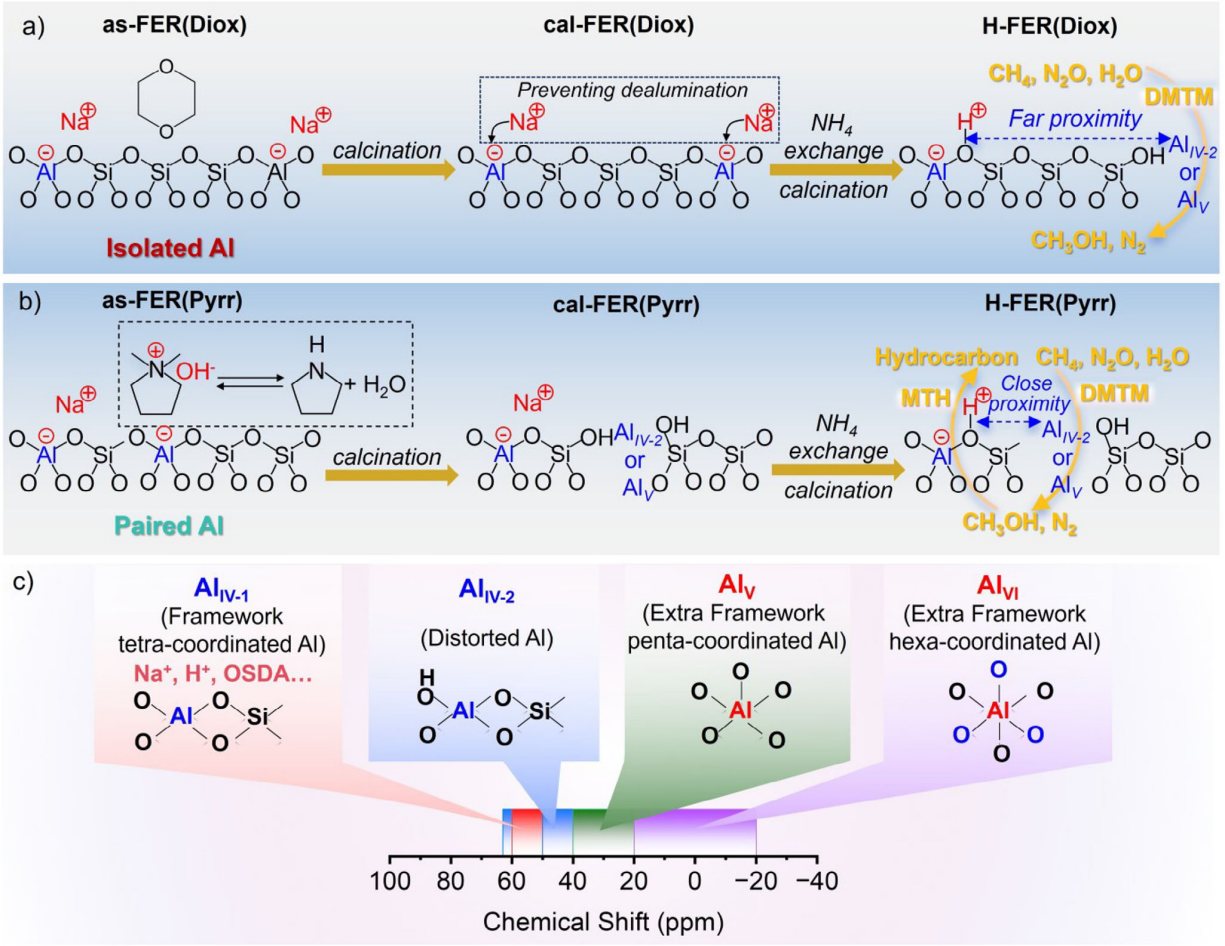
The possible formation route of active Al species on a) FER(Diox) and b) FER(Pyrr) zeolites. c) The Al species and corresponding chemical shift in the ^27^Al MAS NMR spectrum.

The topological structure of homemade FER zeolites was confirmed by X‐ray diffraction (XRD) patterns (Figure S1). The content of dioxane and pyrrolidine was evaluated using thermogravimetric differential thermal analysis (TG‐DTA) (Figure S2), revealing weight losses of approximately 7.2% for as‐FER(Diox) and 10.1% for as‐FER(Pyrr). The exothermic peak for as‐FER(Diox) occurred at 413 °C and was lower than that of as‐FER(Pyrr) at 465 °C. Pyrrolidine was more challenging to eliminate as the OSDA was positioned close to the framework Al and the higher thermal stability (Figure S2c,d). A close Si/Al ratio of as‐FER(Diox) and as‐FER(Pyrr) was obtained (Table 1), while the Na/Si ratio of as‐FER(Diox) was 0.12 and much higher than that of as‐FER(Pyrr) 0.03. The results indicated that the negative charge of AlO_4_^−^ for as‐FER(Diox) was primarily balanced by Na^+^, and the role of the nonionic solution dioxane was pore filling, which was similar to alcohols in the synthesis of ZSM‐5.^[^ ^20^ ^]^ The typical flake morphology was observed for both FER(Diox) and FER(Pyrr) (Figure S3). All elements were uniformly distributed, and no transition or noble metals were observed based on the energy‐dispersive spectroscopy (EDS) mapping images (Figure S4). The narrow peaks observed in the ^27^Al magic angle spinning nuclear magnetic resonance (MAS NMR) spectra of as‐FER(Diox) and as‐FER(Pyrr) indicated that all detectable aluminum atoms were in the framework in a tetra‐coordinated state (Al_IV‐1_) (Figure 1a,d). To identify the Al sites, the ^27^Al multiple‐quantum magic angle spinning (MQMAS) NMR spectra of as‐FER(Diox) and as‐FER(Pyrr) were scanned in different slices along the F1 dimension.^[^ ^28^, ^29^ ^]^ Considering the centers of gravity in the F1 and F2 dimensions of the MQMAS NMR spectra, the T1, T2, T3, and T4 sites exposed isotropic chemical shifts (*δ*_iso_) at approximately 60, 50, 54, and 57 ppm, respectively.^[^ ^28^ ^]^ Some differences between individuals regarding the *δ*_iso_ of T sites are worth pointing out. The proportion of Al atoms at corresponding T sites was calculated based on deconvoluted results and the area of fitting peaks. Specifically, 64% of Al atoms of as‐FER(Diox) were located at T4 sites, which were situated within the FER cage in the 8‐ring channels and shared a 6 R with T2 sites (Figures 1b and S5). 54% of Al atoms occupied T3 sites for as‐FER(Pyrr), where T3 sites were in the 10‐ring channels and shared a 10 R with T1 and T2 sites (Figures 1e and S5). A reasonable speculation is the different sizes of cations. In the case of as‐FER(Diox) zeolite, dioxane is neutral and would be mainly located at 10 R due to the size, resulting in fewer Al atoms located at 10 R, which consists of T1, T2, and T3 sites. Framework Al atoms in as‐FER(Diox) should be balanced by Na^+^, which mainly appeared in 8 R due to its small size. Considering that 10 R would be primarily occupied by dioxane, a preferential population at T4 sites in 8 R is achieved. On the other hand, for as‐FER(Pyrr), both pyrrolidine and Na^+^ can balance the charge of AlO_4_^−^. Hence, framework Al atoms would preferentially populate in 10 R due to the size of pyrrolidine and the low Na/Si ratio in as‐FER(Pyrr). The arrangement of Al atoms in the framework of as‐synthesized FER zeolites was analyzed by ^29^Si MAS NMR spectra (Figure 1c,f). Based on the deconvolution results, 8% of Q^4^(2Al) was obtained by as‐FER(Pyrr), which was higher than 3% observed in as‐FER(Diox).^[^ ^37^, ^38^ ^]^ The results suggest that as‐FER(Pyrr) contained a greater number of Al pairs than as‐FER(Diox). The presence of paired Al in the as‐synthesized sample may lead to proximity between oxidative sites (Al_IV‐2_ or Al_V_) and acidic sites (Si(OH)Al) after calcination, NH_4_‐exchange, and subsequent calcination (Scheme 1), thereby affecting the occurrence of tandem reactions.^[^ ^11^, ^29^ ^]^ Conversely, isolated Al in the as‐synthesized sample may result in more distant bifunctional sites after calcination, NH_4_‐exchange, and further calcination (Scheme 1), thus limiting the conversion of methanol to hydrocarbons at the acidic sites.^[^ ^11^, ^29^ ^]^

**Table 1:** Chemical composition and acid amount of FER zeolites.

	Chemical compositions			Acid amount (mmol g^−1^)/temperature (°C)^a)^		
Sample	Si/Al^b)^	Na/Si^c)^	OSDA/Si^d)^	Weak	Medium	Strong
as‐FER(Diox)	10.0	0.12	0.05	–	–	–
cal‐FER(Diox)	10.0	0.12	–	0.43/176	1.38/255	–
H‐FER(Diox)	10.0	–	–	0.80/184	0.35/284	0.76/425
as‐FER(Pyrr)	9.3	0.03	0.09	–	–	–
cal‐FER(Pyrr)	9.3	0.03	–	0.33/174	0.95/228	0.77/407
H‐FER(Pyrr)	9.3	–	–	0.53/175	0.51/218	0.84/415
H‐FER(Tosoh)	9.0	–	–	1.11/195	0.73/350	0.59/466

^a)^ By NH_3_‐TPD; the weak, medium, and strong acid amounts were fitted at approximately 150–250, 250–400, and 400–600 °C, respectively.

^b)^ By ICP‐AES.

^c)^ By AAS.

^d)^ By TG‐DTA.

**Figure 2:**
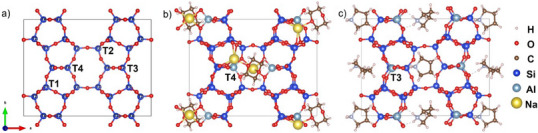
a) The location of four inequivalent T sites in FER zeolite. b) DFT‐predicted preferential Al distribution at T4 sites in FER using 1,4‐dioxane as the pore‐filling agent with Na cations. c) DFT‐predicted preferential Al distribution at T3 sites in pyrrolidinium‐templated FER.

Furthermore, the binding energy predicted by DFT calculations indicated the Al distribution in FER zeolite prepared using dioxane and pyrrolidine.^[^ ^39^ ^]^ FER contains 10‐ring channels that run parallel to the crystallographic [001] direction, intersecting with 8‐ring channels that are parallel to the [010] direction (Figure S5). A unit cell includes four inequivalent tetrahedral (T) sites. T1, T2, and T3 reside in 10‐ring channels, while all T‐sites (T1–T4) lie on the FER cage surface (Figure 2a).^[^ ^40^ ^]^ OSDA and Na^+^ quantities per unit cell were determined from as‐FER(Diox) and as‐FER(Pyrr) compositions (Table S1), enabling DFT modeling of framework Al positions (Figure S6). The minimum relative energy was obtained by as‐FER(Diox) at the T4 site, indicating that Al was preferentially distributed at T4 sites (Figures 2b and S6c). This result was consistent with experimental data derived from the ^27^Al MQMAS NMR spectrum (Figure 1b) and literature that utilized dioxane as the pore‐filling agent.^[^ ^28^ ^]^ In the case of as‐FER(Pyrr), the effect of sodium was disregarded in the DFT calculation due to the relatively low sodium content, and pyrrolidine molecules as the strong templating in the crystallization of FER were accommodated in two sites, one within the ferrierite cavity, and another in the widest section of the 10 R channels.^[^ ^34^ ^]^ The minimum relative energy was obtained by as‐FER(Pyrr) at the T3 site (Figures 2c and S6d), which also agreed with the experimental data based on the ^27^Al MQMAS/MAS NMR spectra that Al preferentially distributed in the T3 site in as‐FER(Pyrr) (Figure 1e).

### Reaction Performance of As‐Synthesized FER Zeolites

**Figure 3:**
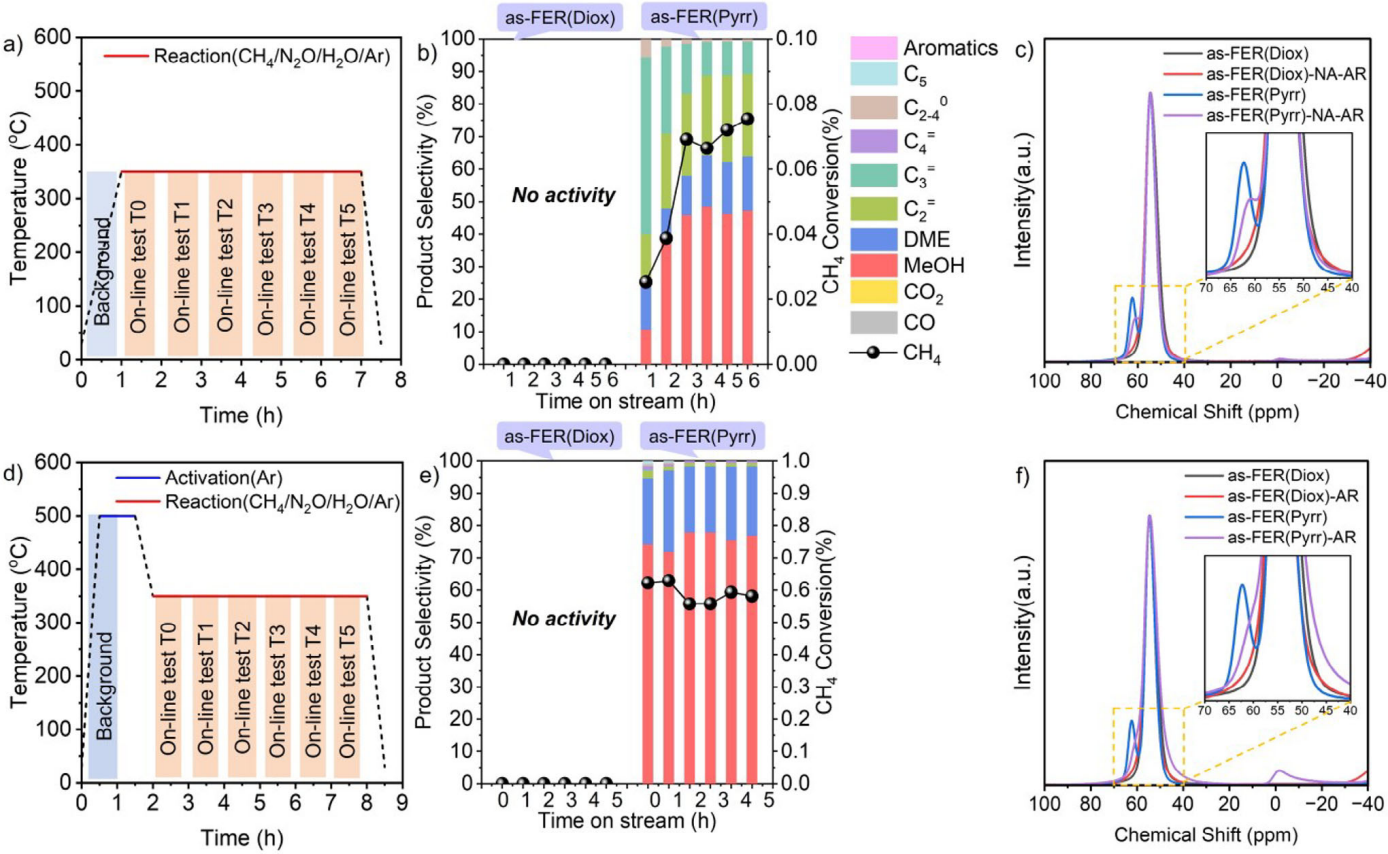
a) Temperature program of the reaction at 350 °C without activation, b) comparison of the reaction performance of as‐FER(Diox) and as‐FER(Pyrr) at 350 °C without activation, c) comparison of the ^27^Al MAS NMR spectra of fresh and spent as‐FER(Diox) and as‐FER(Pyrr) after reaction at 350 °C without activation. d) Temperature program of the reaction at 350 °C after activation at 500 °C for 1 h, e) comparison of the reaction performance of as‐FER(Diox) and as‐FER(Pyrr) at 350 °C after activation at 500 °C for 1 h, f) comparison of the ^27^Al MAS NMR spectra of fresh and spent as‐FER(Diox) and as‐FER(Pyrr) after reaction at 350 °C with activation at 500 °C for 1 h.

Because the normal tetra‐coordinated Al (Al_IV‐1_) in the framework was present in both as‐FER(Diox) and as‐FER(Pyrr), the activity of Al_IV‐1_ in the direct oxidation of methane‐to‐methanol was examined. To protect the framework of Al atoms, the temperature program was settled without activation, and the reaction temperature was directly increased to 350 °C (Figures 3a and S7). No reaction occurred on as‐FER(Diox). However, both the conversion of methane‐to‐methanol (DMTM) and the reactions of methanol‐to‐olefins (MTO) or methanol‐to‐hydrocarbons (MTH) happened on as‐FER(Pyrr) (Figures 3b and S8). Notably, the CH_4_ conversion of as‐FER(Pyrr) increased from around 0.02 to 0.08% after 6 h of reaction at 350 °C, indicating the gradual formation of active Al species during the reaction process, as described in our previous work using the original NH_4_‐type commercial FER zeolite.^[^ ^29^ ^]^ The ^27^Al MAS NMR spectra of the fresh and spent samples were compared in Figure 3c. Almost no difference was observed between the fresh and spent as‐FER(Diox) samples. However, a noticeable variation was detected between the fresh and spent as‐FER(Pyrr) samples. Specifically, the intensity of the band at around 62 ppm assigned to the T1 site decreased after the reaction, and the intensity of the band at 45–50 ppm contributed to distorted tetra‐coordinated Al species (Al_IV‐2_) slightly increased. The results suggested that active Al species were generated from the normal Al_IV‐1_ to distorted Al_IV‐2_, consistent with our recent work.^[^ ^29^ ^]^ In addition, pyrrolidine as the OSDA can be partially removed at 350 °C, which was beneficial for forming Al_IV‐2_, being evidenced by the TG‐DTA curves (Figure S2). On the other hand, Na cations in as‐FER(Diox) resisted dealumination, resulting in the absence of active Al species formation. This behavior was similar to that observed in the Na‐type Fe‐AEI zeolite reported in our recent work^[^ ^41^ ^]^ and our previous study proposed that alkali metal ions, such as Na^+^ and K^+^, can be artificially introduced to inhibit dealumination.^[^ ^29^ ^]^ When an activation step at 500 °C for 1 h was implemented before the reaction at 350 °C for both as‐FER(Diox) and as‐FER(Pyrr) (Figure 3d), the reaction performance, including the formation rate and selectivity of methanol for as‐FER(Pyrr), was significantly improved compared to the case without activation (Figures 3e and S9). However, there was still no activity observed for as‐FER(Diox) (Figure 3e). By comparing the ^27^Al MAS NMR spectra of the fresh and spent samples, the intensity of the band at 40–50 ppm was greatly enhanced for as‐FER(Pyrr), while the sharp peak at 62 ppm shifted to a shoulder at 60–65 ppm, indicating the presence of the Al_IV‐2_ (Figure 3f). In contrast, no significant changes were observed in as‐FER(Diox) due to the resistance of Na cations.^[^ ^41^, ^42^ ^]^ The results provide a comprehensive explanation of the processes and conditions necessary for the formation of active Al species as oxidative sites.

### Comparison of Calcined FER Zeolites from Characterization to Reaction Performance

**Figure 4:**
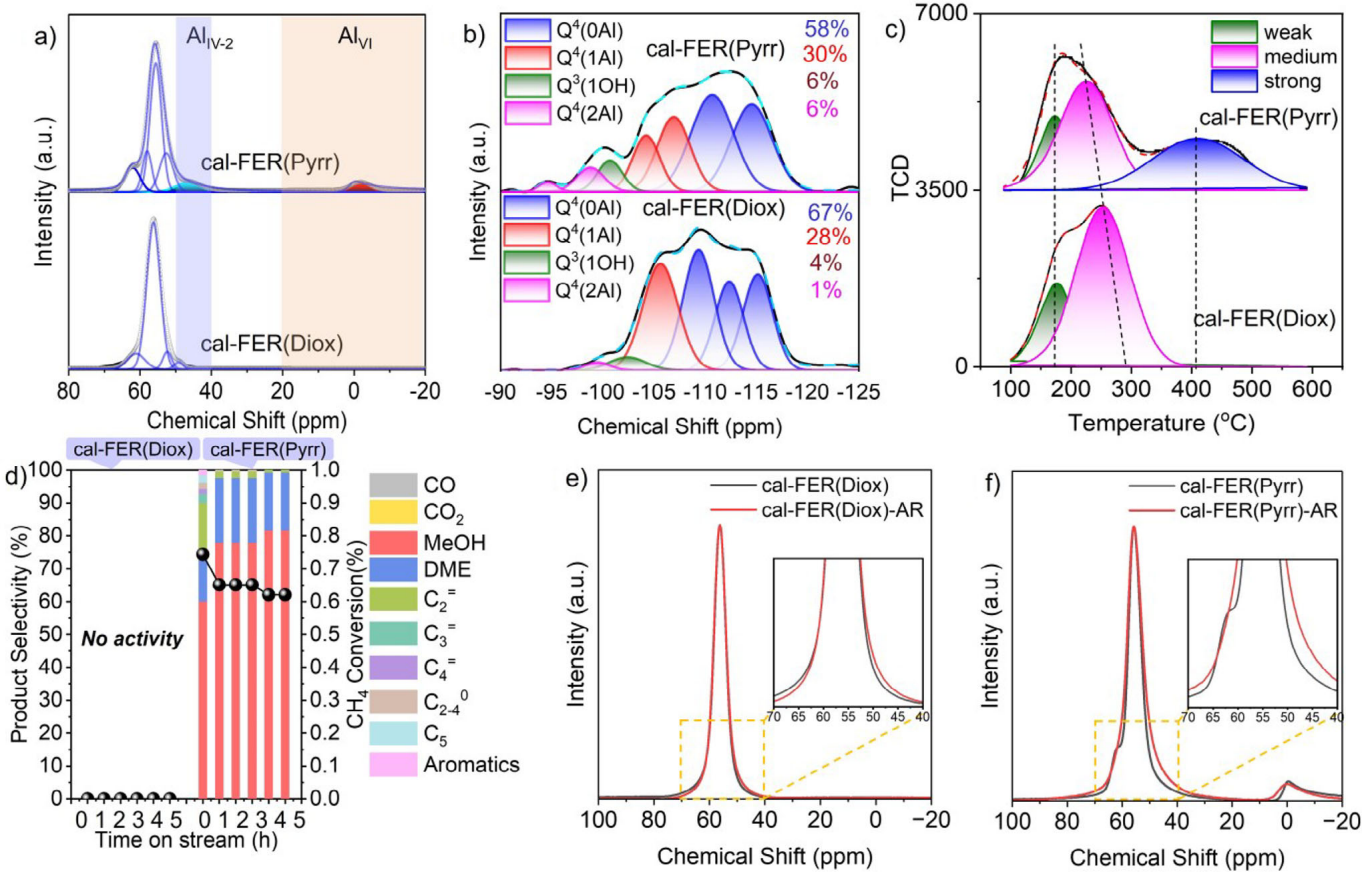
a) Compare the ^27^Al MAS NMR spectra, b) deconvolution of ^29^Si MAS NMR spectra, c) NH_3_‐TPD results of cal‐FER(Diox) and cal‐FER(Pyrr). d) A comparison of the reaction performance of cal‐FER(Diox) and cal‐FER(Pyrr) at 350 °C after activation at 500 °C for 1 h. Compare the ^27^Al MAS NMR spectra of fresh and spent e) cal‐FER(Diox) and f) cal‐FER(Pyrr).

Furthermore, calcined samples were prepared by subjecting the as‐synthesized samples to calcination at 550 °C for 10 h to remove the PFA or OSDA. As displayed in Figure 4a, the ^27^Al MAS NMR spectrum of cal‐FER(Diox) exhibited only a slight difference compared to as‐FER(Diox) (Figures 4a and S10a). In contrast, significant differences were observed in the bands at (70–60, 50–40), 40–20, and 20‐(‐20) ppm for cal‐FER(Pyrr) (Figure 4a), indicating the formation of Al_IV‐2_, Al_V_, and hexa‐coordinated Al (Al_VI_) species (Scheme 1). The deconvolution of the ^29^Si MAS NMR spectrum for cal‐FER(Pyrr) revealed a higher proportion of Q^4^(2Al) than cal‐FER(Diox) (Figure 4b). In addition, dioxane acted as the PFA, and the negative charge of AlO_4_^−^ was balanced by Na^+^. After calcination, dioxane was removed, but Na cations remained in cal‐FER(Diox). These factors contributed to the trace amount of BAS and bridged hydroxyl Si(OH)Al (Figures 4c, S10, and Table 1). Consequently, no catalytic activity was observed for cal‐FER(Diox) in the methane oxidation reaction at 350 °C after activation at 500 °C for 1 h (Figures 4d and S11), and no significant variation was detected between fresh and spent samples in ^27^Al MAS NMR spectra (Figure 4e). In contrast, a notable amount of Brønsted acid sites (BAS) and Lewis acid sites (LAS) were present in cal‐FER(Pyrr) due to the removal of pyrrolidine, which generated H^+^, and the low content of Na (Figures 4c, S10, and Table 1). As a result, the reaction performance of cal‐FER(Pyrr) surpassed that of as‐FER(Pyrr) in terms of the formation rates of methanol and hydrocarbons (Figures 3, 4, and S11). Furthermore, the ^27^Al MAS NMR spectra of fresh and spent cal‐FER(Pyrr) (cal‐FER(Pyrr)‐AR) showed variation in bands at 70–60 and 50‐40 ppm (Figure 4f). When comparing the product distribution of as‐ and cal‐FER(Pyrr), the selectivity of hydrocarbon increased from 5% to 28%. The presence of sodium cations in the zeolite hindered the conversion of methanol to hydrocarbons. The loading of sodium ions through ion exchange with FER(Pyrr) zeolite, along with the observed decrease in CH_4_ conversion, methanol formation rate, hydrocarbon formation rate, and hydrocarbon selectivity with increasing Na content, confirmed the roles of Na cations and BAS in the methane oxidation reaction (Figure S12). The formation of active Al species in the calcined samples is illustrated in Scheme 1. Additionally, the reaction performance of calcined FER(Diox) and FER(Pyrr) in the conversion of methanol‐to‐hydrocarbons was investigated (Figure S13). As anticipated, cal‐FER(Diox) exhibited no activity due to the low acid content, while cal‐FER(Pyrr) demonstrated activity but was rapidly deactivated because of its 2D topological structure.

### Comparison of H‐Type FER Zeolites from Characterization to Reaction Performance

**Figure 5:**
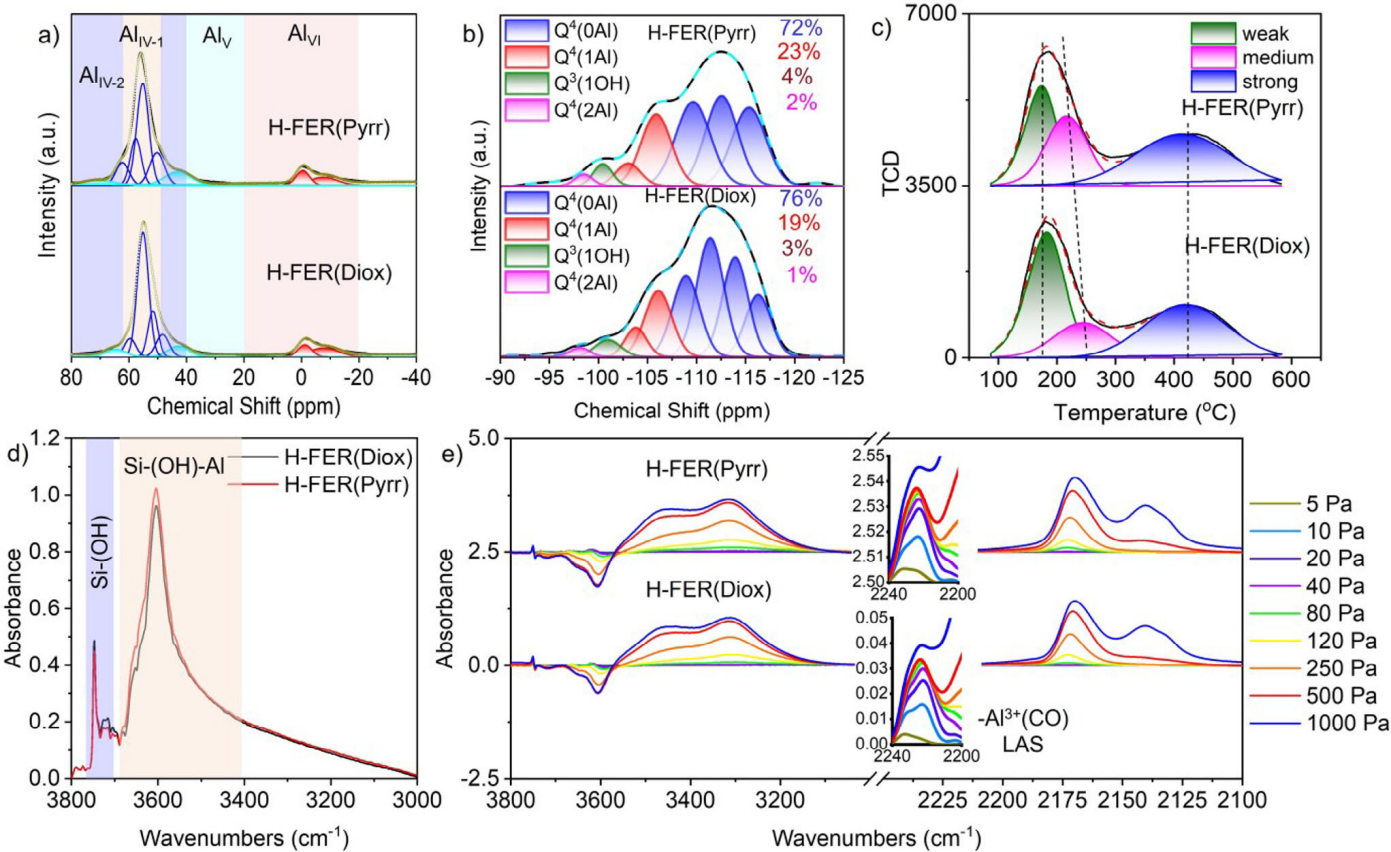
a) ^27^Al MAS NMR spectra of H‐FER(Diox) and H‐FER(Pyrr). b) Deconvolution of ^29^Si MAS NMR spectra, c) NH_3_‐TPD profiles, d) hydroxyl stretching vibration, and e) CO adsorption FTIR spectra of H‐FER(Diox) and H‐FER(Pyrr) at −120 °C after activation at 500 °C for 1 h.

Afterward, Na cations were replaced by proton (H^+^) through NH_4_^+^ exchange and calcination (Scheme 1). Similar chemical compositions and textural properties were observed between H‐FER(Diox) and H‐FER(Pyrr) (Figures S14, Table 1 and S2). The ^27^Al MAS NMR spectra of H‐FER(Diox) and H‐FER(Pyrr) nearly overlapped (Figure 5a). Meanwhile, a similar proportion of Al*_I_*_V‐1_(75%), Al_IV‐2_(12%), Al_V_(0%), and Al_VI_(13%) was obtained for both H‐FER(Diox) and H‐FER(Pyrr) (Table 2 and Scheme 1). However, the ^27^Al MQMAS NMR spectra displayed different results (Figure S15a,b). Among the total framework Al, the highest proportion of 45% Al was located at the T4 site for H‐FER(Diox), while 36% Al was situated at the T3 site for H‐FER(Pyrr) (Table 2). The deconvolution of ^29^Si MAS NMR spectra revealed a slightly higher amount of Q^4^(2Al) for H‐FER(Pyrr) (Figures 5b and S15). It is noteworthy that the proportion of Q^4^(2Al) for H‐FER(Pyrr) decreased from 8% in as‐FER(Pyrr) to 1%, indicating that a large part of the shed Al, or the potential oxidative Al species, originated from Al pairs (Scheme 1).

**Table 2:** Al distribution of FER zeolites identified by ^27^Al MAS NMR.

	Al distribution (%)				Framework Al distribution (%)			
	Al_IV‐1_	Al_IV‐2_	Al_V_	Al_VI_	T1	T2	T3	T4
Sample	63∼50 ppm	∼63, 50∼40 ppm	40∼20 ppm	20∼(−20) ppm	60 ppm	50 ppm	54 ppm	57 ppm
as‐FER(Diox)	100	0	0	0	7	9	20	64
cal‐FER(Diox)	100	0	0	0	10	9	17	64
H‐FER(Diox)	75	12	0	13	7	9	14	45
as‐FER(Pyrr)	100	0	0	0	16	23	54	7
cal‐FER(Pyrr)	91	4	0	5	11	17	48	12
H‐FER(Pyrr)	73	15	0	12	9	14	36	15
H‐FER(Tosoh)	94	4	0	2	8	3	11	71

**Figure 6:**
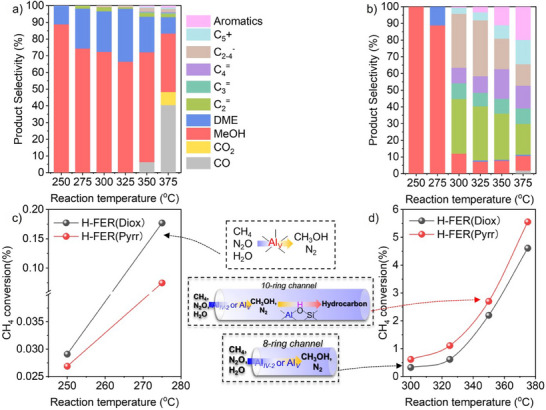
Product distribution of a) H‐FER(Diox) and b) H‐FER(Pyrr) at 250–375 °C. A comparison of the CH_4_ conversion for H‐FER(Diox) and H‐FER(Pyrr) at c) 250–275 °C and d) 300–375 °C. Reaction conditions: 100 mg catalyst, CH_4_/N_2_O/H_2_O/Ar = 10/10/2/3 ml min^−1^, WHSV = 15 000 ml g^−1^ h^−1^, TOS = 0.16 h.

Interestingly, the NH_3_‐TPD profiles and hydroxyl vibration curves of H‐FER(Diox) and H‐FER(Pyrr) nearly overlapped (Figure 5c,d). The slight difference between H‐FER(Diox) and H‐FER(Pyrr) was reflected in the LAS measured by CO adsorption FTIR spectra at −120 °C (Figure 5e). The intensity of the band at 2230 cm^−1^ ascribed to LAS for H‐FER(Pyrr) was stronger than that of H‐FER(Diox). We did not assert that the active sites for the conversion of methane‐to‐methanol were related to LAS, as the higher activity was achieved by H‐FER(Diox) at 250 °C with fewer LAS than H‐FER(Pyrr) (Figures 6, S16, and S17). In addition, in our latest work, FER zeolite from Zeolyst after calcination at 850 °C showed higher activity than that at 550 °C.^[^ ^29^ ^]^ However, the intensity of the band at 2230 cm^−1^ for H‐FER(Zeolyst)‐850 was almost identical to that of H‐FER(Zeolyst)‐550 (Figure S18). When the reaction temperatures ranged from 250 to 275 °C, methanol and dimethyl ether (DME) were the primary products for both H‐FER(Diox) and H‐FER(Pyrr) (Figures 6, S16, and S17). Consequently, it was appropriate to compare the activity of the direct oxidation of methane‐to‐methanol. The CH_4_ conversion and CH_3_OH formation rate for H‐FER(Diox) were higher than those of H‐FER(Pyrr) (Figure 6c). We observed that most of the newly formed Al_IV‐2_, Al_V_, and Al_VI_ were derived from the dealumination of framework Al at T4 sites for H‐FER(Diox) and T3 sites for H‐FER(Pyrr) (Table 2). Therefore, we concluded that Al species originating from T4 sites exhibited greater activity than those from T3 sites. It is important to note that more precise comparisons will be necessary through DFT calculations in our future research.

When the reaction temperatures ranged from 300 to 350 °C, the situation became complex due to the tandem reaction of methanol to hydrocarbons at acidic sites, as well as the over‐oxidation of methane and methanol to CO_2_. The initial hydrocarbon selectivity of H‐FER(Pyrr) reached up to 90%, while that of H‐FER(Diox) was less than 10% under the premise of a similar acid amount at 300–350 °C with a favorable carbon balance (Figures 6 and S19–S21; Tables 1 and S3). As emphasized in our recent work, except for the acid amount, the distance between the oxidative sites and the acid sites, as well as the propagation path of methanol, significantly influenced the production of olefins.^[^ ^21^ ^]^ The different hydrocarbon selectivity between H‐FER(Diox) and H‐FER(Pyrr) was primarily attributed to the varying proximity of the oxidative sites (Al_IV‐2_ or Al_V_) to the acidic sites (Si(OH)Al). As illustrated in Scheme 1a,b, as‐FER(Pyrr) contained a higher proportion of Al pairs compared to as‐FER(Diox), a finding corroborated by the ^29^Si MAS NMR results shown in Figure 1c,f. The bifunctional sites formed by the Al pairs were located in close proximity, facilitating the tandem conversion of methane to methanol on the oxidative sites, followed by the conversion of methanol to hydrocarbons on the acidic sites. In contrast, the bifunctional sites formed by isolated Al atoms were situated farther apart, which hindered the subsequent reactions.^[^ ^10^ ^]^ Moreover, the higher CH_4_ conversion of H‐FER(Pyrr) than H‐FER(Diox) at 300–350 °C indicated that the successive MTH reaction could promote the CH_4_ activation (Figure 6d). When H‐FER(Diox) and H‐FER(Pyrr) were employed in the MTH reaction at 350 °C, C_3‐4_^=^ and C_5_ were produced as the main products (Figure S22). However, H‐FER(Diox) was more prone to inactivation than H‐FER(Pyrr), likely due to a greater number of acid sites in H‐FER(Diox) located within 8‐ring channels, as demonstrated by the Al distribution and corresponding T sites (Table 2).

In addition, the commercial FER zeolite from Tosoh (H‐FER(Tosoh)) with the Si/Al ratio of 9 and 71% of Al sitting at T4 sites (Figures S23 and S24; Tables 2 and S2), being higher than 45% for H‐FER(Diox) (Table 2). A similar product distribution of H‐FER(Tosoh) and H‐FER(Diox) was observed at temperatures ranging from 250 to 350 °C, with minimal hydrocarbon (Figures S25–S29). Notably, the CH_4_ conversion and CH_3_OH formation rate for H‐FER(Tosoh) were greater than those for H‐FER(Diox) at 250–350 °C. These results suggested that the Al species with increased activity were located at T4 sites.

**Scheme 2:**
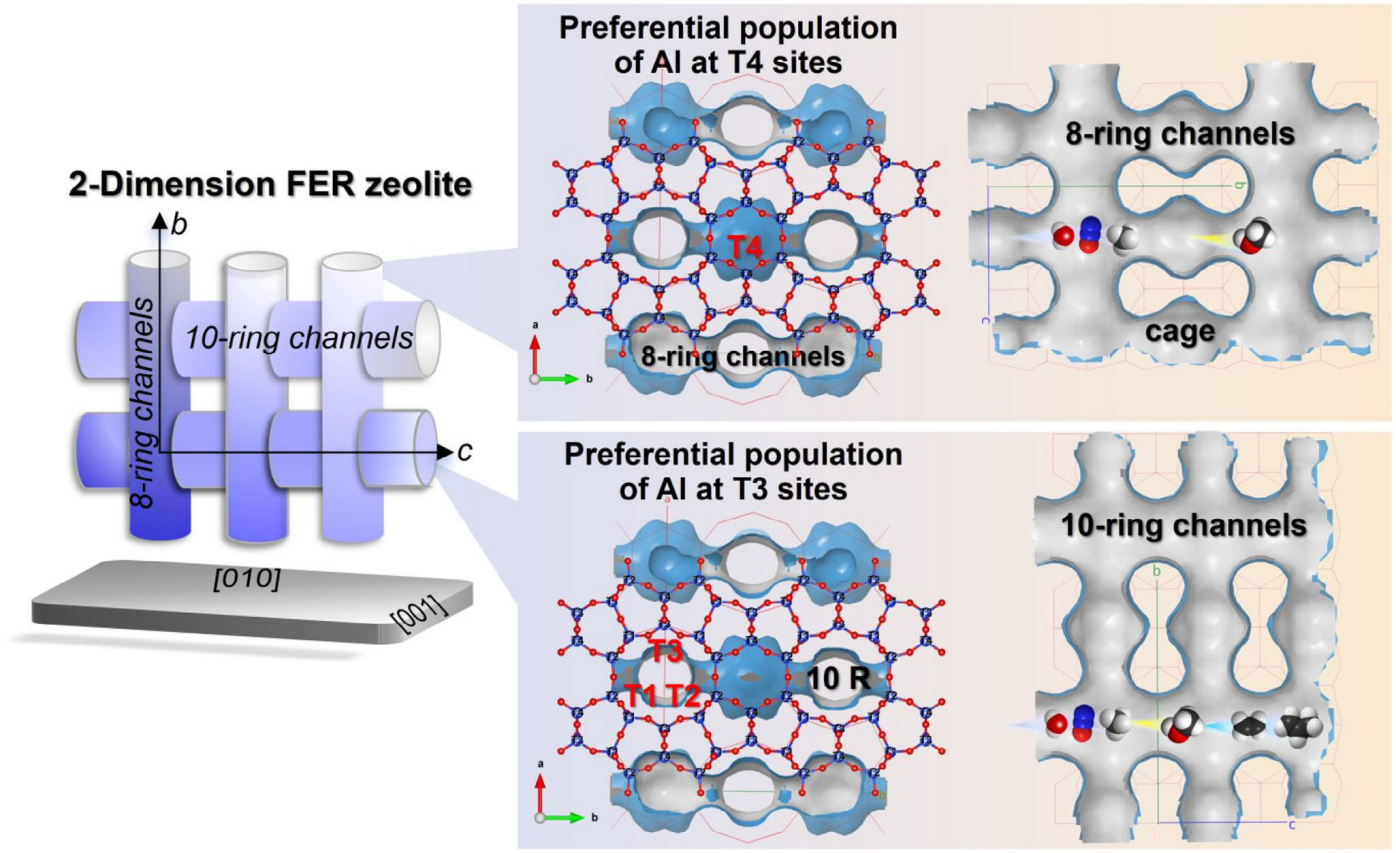
The possible schematic diagram of the preferential population of Al atoms at T4 or T3 sites, specifically within the 8‐ or 10‐ring channels of the plate‐like FER zeolite, and its influence on the reaction performance of methane oxidation.

When the reaction temperature was increased to 375 °C, H‐FER(Diox) and H‐FER(Pyrr) exhibited initial hydrocarbon selectivities of approximately 7% and 89%, respectively (Figures 6 and S30). We attribute the differences in selectivity to the distribution of Al within the zeolite framework, specifically the location of Al atoms. In the case of H‐FER(Diox) zeolite, the majority of the framework Al atoms were situated at T4 sites (Table 2), which are aligned along the *b* axis and feature a short propagation path (Figure 6d and Scheme 2).^[^ ^43^, ^44^ ^]^ Furthermore, the BAS generated by framework Al at T4 sites within the FER cage may be oriented toward the 6 R rather than the 8‐ring channels (Scheme S1). Notably, T4 sites are not positioned at the center of the 8‐ring channels, leading to a low probability of methanol propagation to the BAS (Figure 6d and Scheme S1).^[^ ^28^ ^]^ In contrast, for H‐FER(Pyrr) zeolite, most of the Al atoms were located at T1, T2, and T3 sites, which correspond to the 10‐ring channels along the long *c* axis of the plate‐like FER zeolite. This configuration provides ample opportunities for methanol to interact with acidic sites along the extended propagation path, thereby facilitating the MTH reaction (Scheme 2).

## Conclusion

In this study, we clarified the significance of the Al distribution in FER zeolite on methane oxidation. FER zeolites with different Al distributions were successfully prepared using dioxane and pyrrolidine as PFA and OSDA, respectively. Framework Al atoms were preferentially populated at T4 sites in the FER(Diox) zeolite and at T3 sites in the FER(Pyrr) zeolite, as determined by ^27^Al MQMAS/MAS NMR spectra and DFT calculations. The active distorted tetra‐coordinated Al (Al_IV‐2_) and penta‐coordinated Al (Al_V_) species generally originated from T4 and T3 sites in H‐FER(Diox) and H‐FER(Pyrr), respectively. The higher CH_4_ conversion of H‐FER(Diox) than H‐FER(Pyrr) at 250–275 °C evidenced the higher activity of Al species from T4 sites in the direct oxidation of methane to methanol reaction. Meanwhile, the Al distribution, including the arrangement and location of Al, affected the occurrence of methanol to hydrocarbon as a tandem reaction at 300–375 °C. The Al arrangement referred to the proximity between oxidative sites and acidic sites, and the Al locations involved the propagation pathways of intermediates. A higher proportion of Al atoms situated in the 10‐ring channels was more likely to facilitate the tandem conversion of methane to methanol at the oxidative sites, followed by the conversion of methanol to hydrocarbons at the acidic sites. This work not only deepens our understanding of previous findings but also underscores the importance of Al distribution in FER zeolite for methane oxidation, providing valuable insights and references for future research.

## Supporting Information

The authors have cited additional references within the Supporting Information.^[^ ^45^, ^46^, ^47^, ^48^, ^49^, ^50^ ^]^

## Conflict of Interests

The authors declare no conflict of interest.

## Supporting information



Supporting Information

## Data Availability

The data that support the findings of this study are available from the corresponding author upon reasonable request.
