# Supplementary material for: Aluminum Distribution in Ferrierite Zeolites Influences the Performance of Methane Oxidation
Source: Angew Chem Int Ed Engl. 2025 Jun 25;64(33):e202506023. doi: 10.1002/anie.202506023 (PMC12338448; doi:10.1002/anie.202506023)
Supplement: Supplementary file 1 — Supporting Information [file ANIE-64-e202506023-s001.docx]

**Supporting information**

Aluminum distribution in Ferrierite zeolites influences the performance of methane oxidation

Peipei Xiao,^[a]^ Xiaomin Tang,^[b]^ Hiroto Toyoda,^[a]^ Yilin Wang,^[a]^ Anmin Zheng,^[b,c]^ Lizhuo Wang,^[d]^ Jun Huang,^[d]^ Masato Sawada,^[a]^ Kengo Nakamura,^[a]^ Yong Wang,^[a]^ Hermann Gies,^[a]^ Toshiyuki Yokoi*^[a,e]^

[a] P. Xiao, H. Toyoda, M. Sawada, K. Nakamura, Y. Wang, H. Gies, T. Yokoi

Institute of Integrated Research,
Institute of Science Tokyo,
4259 Nagatsuta, Midori-ku, Yokohama 226-8501, Japan
E-mail: yokoi@cat.res.titech.ac.jp

[b] X. Tang, A. Zheng
State Key Laboratory of Magnetic Resonance Spectroscopy and Imaging, National Center for Magnetic Resonance in Wuhan, Innovation Academy for Precision Measurement Science and Technology,
Chinese Academy of Sciences,
Wuhan, 430071, China

[c] A. Zheng
Interdisciplinary Institute of NMR and Molecular Sciences, Hubei Province for Coal Conversion and New Carbon Materials, School of Chemistry and Chemical Engineering,
Wuhan University of Science and Technology,
Wuhan, 430081, China

[d] L. Wang, J. Huang
School of Chemical and Biomolecular Engineering,
the University of Sydney,
Sydney, New South Wales 2006, Australia

[e] T. Yokoi

iPEACE223 Inc.,
Konwa Building, 1-12-22 Tsukiji, Chuo-ku, Tokyo, 104-0045, Japan

**Catalyst preparation**

The aluminosilicate FER zeolites were synthesized using 1,4-dioxane and pyrrolidine as the PFA and OSDA, respectively, as referred to literature.^[1, 2]^ Specifically, a basic solution with the composition of SiO_2_: 0.05Al_2_O_3_: 0.0625Na_2_O: 1 dioxane or pyrrolidine: 20H_2_O was obtained by dissolving dioxane or pyrrolidine, NaOH, and NaAlO_2_ in deionized water, and colloidal silica was then added to the solution and vigorously stirred at room temperature for 1 h. Finally, the obtained gel was transferred into an autoclave and subjected to crystallization in a tumbling oven (20 rpm) at 165 ^o^C for 7 days. The solid product was collected by centrifugation, washed with deionized water until a near-neutral pH, and dried overnight at 100 ^o^C. The as-synthesized samples were tagged as-FER(Diox) and as-FER(Pyrr). 1,4-Dioxane and pyrrolidine in as-FER(Diox) and as-FER(Pyrr) were removed by calcination at 550 ^o^C for 10 h, resulting in samples named cal-FER(Diox) and cal-FER(Pyrr), respectively. H-FER(Diox) and H-FER(Pyrr) were prepared by exchanging cal-FER(Diox) and cal-FER(Pyrr) with 2.5 mol·L^-1^ NH_4_NO_3_ at 80 ^o^C for 3 h and followed by calcination in air at 550 ^o^C for 5 h.

Commercial FER zeolite was purchased from Tosoh (NH_4_^+^-FER, 720NHA). The sample was calcined at 550 ^o^C for 5 h in air before use and designated as H-FER(Tosoh).

**Catalyst characterization**

XRD pattern was collected on a Rint-Ultima III (Rigaku) using a Cu Kα X-ray source (40 kV, 40 mA).

Elemental analyses of samples were performed on an inductively coupled plasma-atomic emission spectrometer (ICP-AES, Shimadzu ICPE-9000).

The Na content of the products was determined using an atomic absorption spectrometer (AAS, Shimadzu AA-6200).

The amount of PFA or OSDA in the as-synthesized samples was determined by measuring the weight loss from 250 to 800 °C using a thermogravimetric (TG) profile. This analysis was conducted on a thermogravimetric-differential thermal analyzer (TG-DTA, Rigaku Thermo Plus EVO II).

Field-emission scanning electron microscopic (FE-SEM) images of the powder samples were obtained on a SU9000 (Hitachi) microscope operating at 1 kV.

High-angle annular dark field scanning transmission electron microscopy (HAADF-STEM) imaging, integrated differential phase contrast STEM (STEM-iDPC) imaging, and energy dispersive spectrometry (EDS) mapping were conducted using a FEI Themis Z microscope equipped with an XFEG field electron source and a double aberration corrector, operating at 300 keV. The HAADF-STEM images were acquired with a camera length of 115 mm while the beam convergence was 25.1 mrads. The pixel size is 37 pm, and the dwell time is 2 us/pixel. The collection angle of the HAADF detector was set to 48–200 mrads. STEM-EDS elemental maps were acquired with a 4 us/pixel acquisition time using Velox software. High-resolution transmission electron microscopy (HRTEM) images were acquired on the same instrument operated at 300 keV.

Solid-state ^27^Al MAS NMR spectra were measured on a JEOL ECA-600 spectrometer at a resonance frequency of 156.4 MHz using a 4 mm sample rotor with a spinning rate of 15.0 kHz. The ^27^Al chemical shift was referenced to -0.54 ppm of AlNH_4_(SO_4_)_2_·12H_2_O.

^27^Al MQMAS NMR spectra were measured using the same equipment and obtained through a three-pulse sequence employing a z-filter. The lengths of the triple quantum excitation and conversion pulses were optimized to 3.4 and 1.8 μs, respectively, while the length of the selective pulse was set to 15.0 μs. A series of 512 t1 slices were collected using 2000 FIDs with a recycle delay of 0.1 s for each slice. The ^27^Al single pulse MAS spectra were decomposed into three components, and the fitting values, including the averaged isotropic chemical shift δ*_iso_* the width of the Gaussian distribution of δ*_iso_* (ΔCS), and the averaged quadrupolar coupling constant CQ were determined using the ‘‘Dmfit’’ program applying a simple Czjzek model.

The solid-state ^29^Si MAS NMR and ^29^Si CP MAS NMR spectra were acquired using a JEOL ECA-600 spectrometer (14.1 T) equipped with an additional 1 kW power amplifier. The ^29^Si chemical shift was referenced to −34.12 ppm of polydimethylsiloxane (PDMS). The samples were spun at 15 kHz by using a 4 mm ZrO_2_ rotor.

Temperature-programmed ammonia desorption (NH_3_-TPD) profiles were recorded on Multitrack TPD equipment (Japan BEL). Typically, 25 mg of catalyst was pretreated at 600 ^o^C in He (50 mL min^−1^) for 1 h and then cooled to 100 ^o^C. Before the adsorption of NH_3_, the sample was evacuated at 100 ^o^C for 1 h. Approximately 2500 Pa of NH_3_ was allowed to contact the sample at 100 ^o^C for 10 min. Subsequently, the sample was evacuated to remove weakly adsorbed NH_3_ at the same temperature for 30 min. Finally, the sample was cooled to 100 ^o^C and heated from 100 to 600 ^o^C at a ramping rate of 10 ^o^C min^−1^ in a He flow (50 mL min^−1^). A thermal conductivity detector (TCD) was used to monitor desorbed NH_3_. The amount of acid sites was determined by the fitting peak area of the profiles.

Nitrogen adsorption and desorption measurements were conducted at -196 °C on a Belsorp-mini II (MicrotracBEL) to obtain information on micro- and mesoporosities.

The acidity of FER zeolites was analyzed via FTIR analysis using CO as the probe molecule. The samples were pressed into a self-supporting disk (20 mm diameter, 30−60 mg) and placed in an IR cell attached to a closed-gas circulation system. The TGS detector was used. The samples were degassed under vacuum conditions at 500 °C for 1 h to remove adsorbed water. The adsorption of CO with different pressures (5-1000 Pa) was performed at -120 ^o^C.

**Computational details**

To investigate the influence of dioxane and pyrrolidine on framework aluminum distribution, periodic density functional theory (DFT) optimizations were performed for FER(Diox) and FER(Pyrr) complexes using the Vienna Ab-initio Simulation Package (VASP).^[1]^ The FER framework was obtained from the IZA database, and aluminosilicate zeolites were generated by replacing four Si atoms in a unit cell with four Al atoms at the same specific crystallographic T site (T1, T2, T3, and T4, respectively). It should be noted that pyrrolidine exists in its protonated state (pyrrolidinium) to balance the negative charge from the framework Al. In the case of as-FER(Diox), dioxane (primarily a pore-filling agent) coexists with Na⁺ ions, where Na⁺ dominates Al distribution control. Consistent with thermogravimetric results, the unit cell contains 2 dioxane molecules and 4 Na⁺ ions. Plane waves were constructed with a kinetic energy cutoff of 400 eV using the projector augmented wave (PAW) method. ^[2-3]^ The 1×1×2 k-point mesh is used and centered at the Γ point. The calculation was performed using the Perdew-Burke-Ernzerh (PBE) generalized gradient approximation (GGA)^[4]^ in conjunction with Grimme’s D3 correction^[5]^ to account for dispersion interaction. The self-consistent field (SCF) electronic energies and atomic forces were converged to 10^-5^ eV and less than 0.02 eV/Å, respectively. The molecular size of dioxane and pyrrolidine was calculated using the Multiwfn package ^[6]^ and visualized by the VMD package ^[7]^.

**Catalytic tests**

**Direct oxidation of methane reaction**

In a typical test, 100 mg of granular catalyst (particle size 500−1000 μm) was loaded into a quartz tube with an inner diameter of 4 mm, which was then placed in an electric tube furnace. The catalyst was pretreated at 500 °C for 1 h in Ar flow. The reaction was conducted at 350 °C in a flowing gas mixture of methane (CH_4_), nitrous oxide (N_2_O), water vapor (H_2_O), and argon (Ar) with flow rates of 10, 10, 2, and 3 mL·min^-1^, respectively. The outlet gas, containing the products as well as unreacted CH_4_ and N_2_O, was analyzed using two online gas chromatographs (GC; GC-2014, Shimadzu). One of the GCs was equipped with a Shin Carbon ST 50/80 packed column (Agilent Technologies; inner diameter 3 mm, length 6 m) and a thermal conductivity detector (TCD). This GC-TCD was used to detect hydrogen (H_2_), N_2_O, carbon monoxide (CO), carbon dioxide (CO_2_), and CH_4_. The other GC was fitted with a Porapak Q 80/100 packed column (Agilent Technologies; inner diameter 3 mm, length 6 m), a flame ionization detector (FID), and a methanizer. The GC-FID was employed to analyze CH_4_, as well as the produced methanol (MeOH), dimethyl ether (DME), alkanes, and alkenes. The yield of each carbon-containing product was calculated based on the number of carbon atoms. In this study, methane conversion was defined as the total amount of obtained products and was calculated as follows:

*C*_CH4_ =


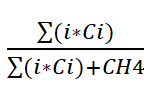

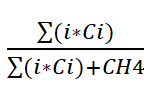


where *C*_CH4_ is the CH_4_ conversion, *i* is the number of carbon atoms in product *C_i_*, Σ(*i*C_i_*) is the total amount of carbon of all the products, and *CH4* is the amount of CH_4_ detected at the same time.

The N_2_O conversion was calculated as follows:

*C*_N2O_ =


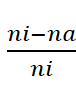

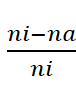


where *C*_N2O_ is the N_2_O conversion, n*_i_* is the initial N_2_O molar weight, n_a_ is the N_2_O molar weight after reaction.

Note that, the calculation methods of CH_4_ and N_2_O conversion were different, resulting in the two values not being equal.

The product selectivity was calculated as follows:

*S*_Ci_ =


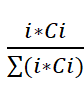

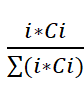


where *S*_Ci_ is the selectivity of the product C_i_, Σ(*i*C_i_*) is the total amount of carbon of all the products.

The product yield was calculated as follows:

*Y*_Ci_ =


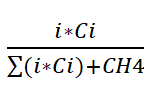

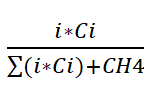


where *Y*_Ci_ is the yield of the product C_i_, Σ(*i*C_i_*) is the total amount of carbon of all the products, and *CH4* is the amount of CH_4_ detected at the same time.

The product formation rates were calculated as follows:

*r*_Ci_ = *Y*_Ci *_*F*_CH4_/*m_cat_*

where *r*_Ci_ is the formation rate of product C*_i_*, *F*_CH4_ is the initial flow rate of CH_4_, *m_cat_* is the mass of catalyst. *r_hydrocarbons_*= 2*(*r_C2_^=^ + r_C2_^-^*) + 3*(*r_C3_^=^ + r_C3_^-^*) + 4*(*r_C4_^=^ + r_C4_^-^*) + 5*(*r_C5_^=^ + r_C5_^-^*).

**Methanol to hydrocarbons (MTH) reaction**

The MTH reaction was performed using a fixed-bed reactor connected to an online gas chromatograph (GC-2014, Shimadzu) equipped with an HP-PLOT/Q capillary column and a flame ionization detector. The 50/80 mesh zeolite pellets without a binder were placed in a 6 mm quartz tubular flow reactor. The pretreatment was conducted at 500 °C for 30 min under Ar (20 mL·min^−1^). After the pretreatment, the reactor was cooled to 350 °C, and the MTH reaction commenced. The pressure of methanol was set at 5 kPa with Ar gas as the carrier; the weight-to-feed ratio (W/F) for methanol was set at 68 g·h·mol^−1^. The product stream was analyzed using a system that automatically injected the product into a gas chromatograph connected directly to the outlet of the reactor via a heated transfer line.

The conversion and selectivity were calculated as follows:

𝐶𝑜𝑛𝑣𝑒𝑟𝑠𝑖𝑜𝑛 𝑜𝑓 𝑚𝑒𝑡ℎ𝑎𝑛𝑜𝑙 [%] = 1 ‒ × 100


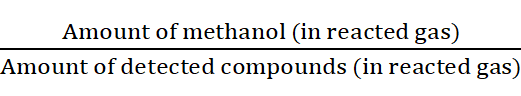

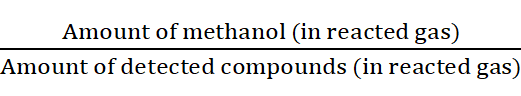


𝑃𝑟𝑜𝑑𝑢𝑐𝑡 𝑠𝑒𝑙𝑒𝑐𝑡𝑖𝑣𝑖𝑡𝑦 [%] = × 100


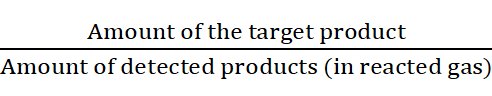

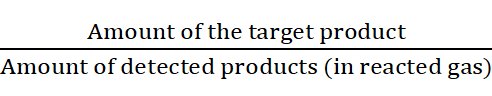

**Figure S1** XRD patterns of FER zeolites.


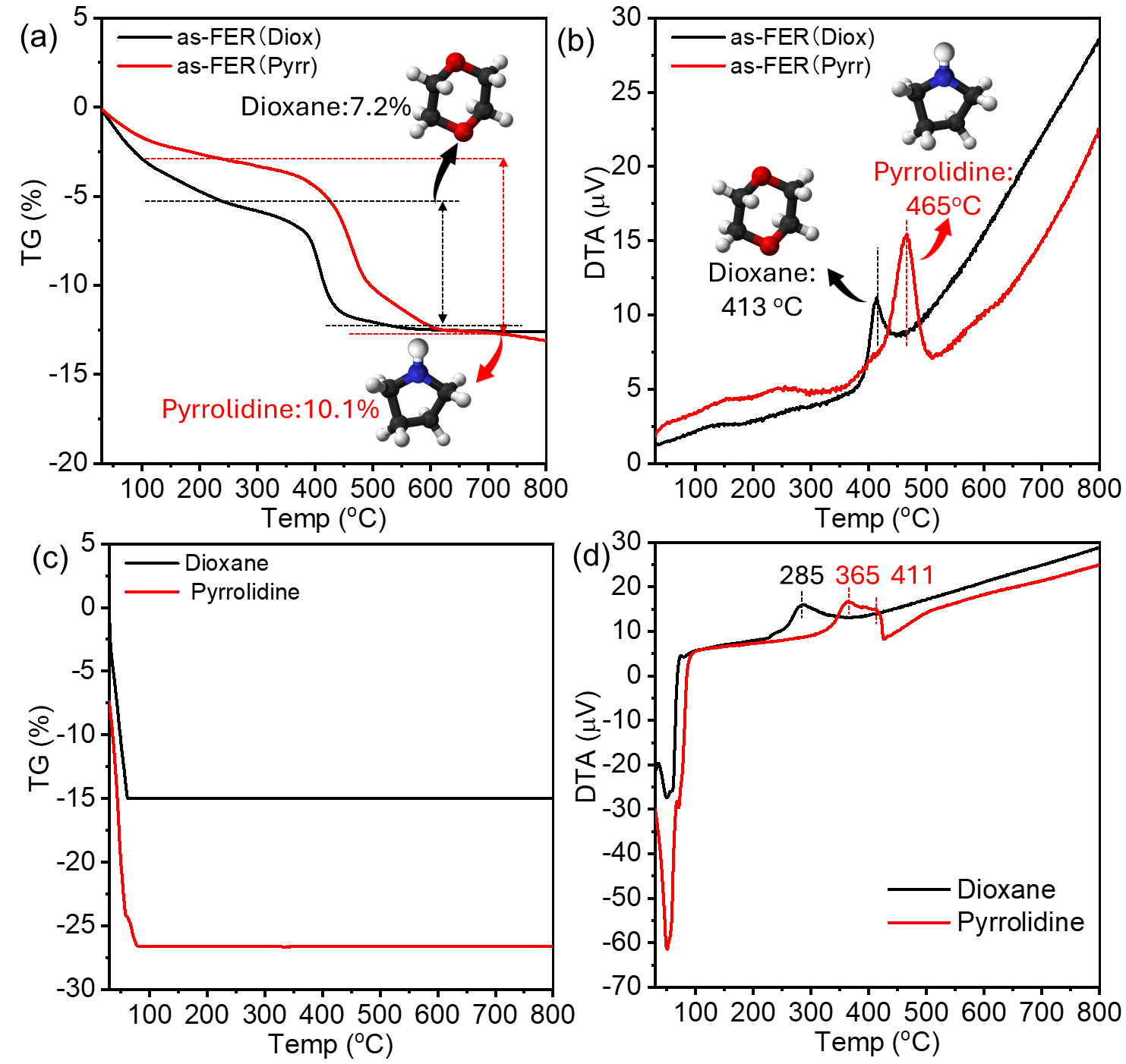


**Figure S2** (a) TG analysis and (b) DTA curves of the as-synthesized FER zeolites. (c) TG analysis and (d) DTA curves of pure dioxane and pyrrolidine.


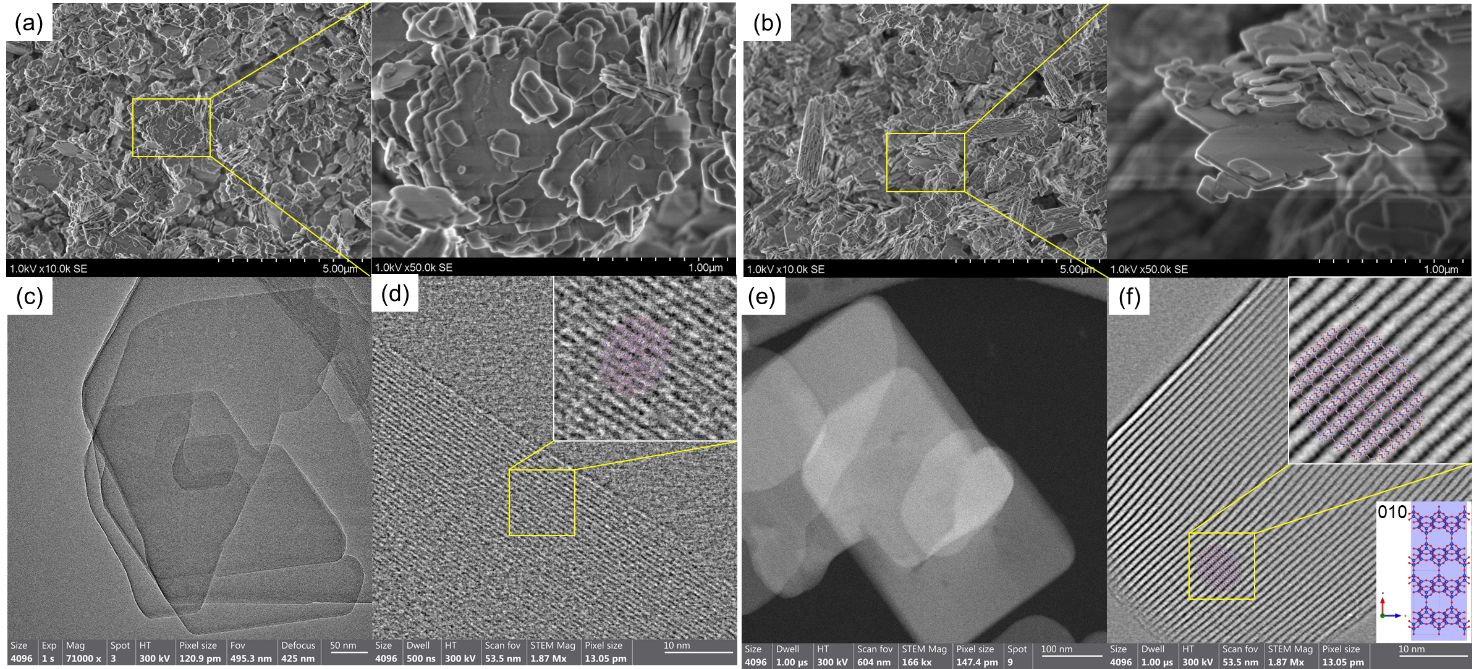


**Figure S3** SEM images of (a) as-FER(Diox) and (b) as-FER(Pyrr). HAADF-STEM images of (c) as-FER(Diox) and (e) as-FER(Pyrr). iDPC-STEM images of (d) as-FER(Diox) and (f) as-FER(Pyrr)
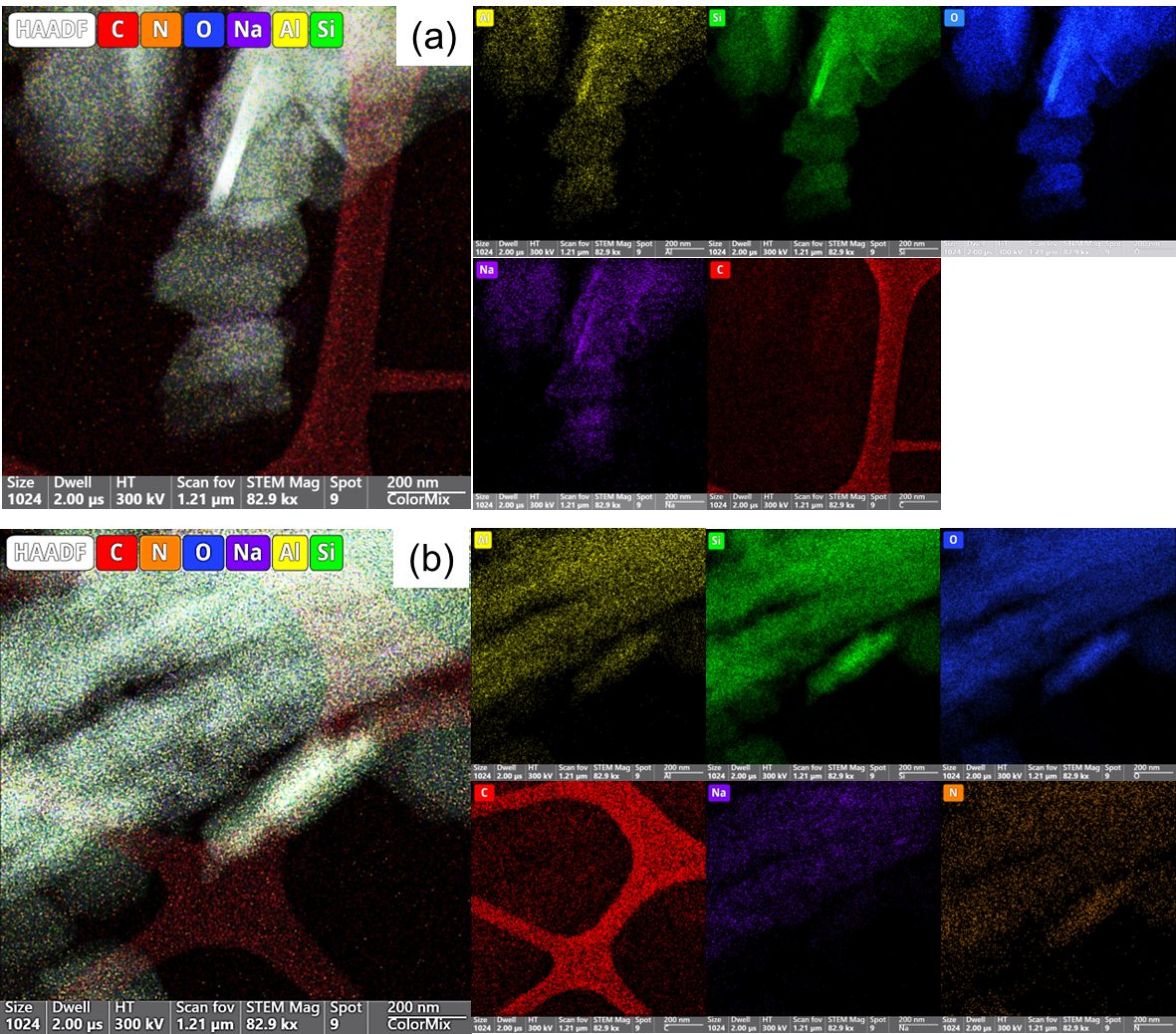


**Figure S4** EDS elements mapping images of (a) as-FER(Diox) and (b) as-FER(Pyrr).


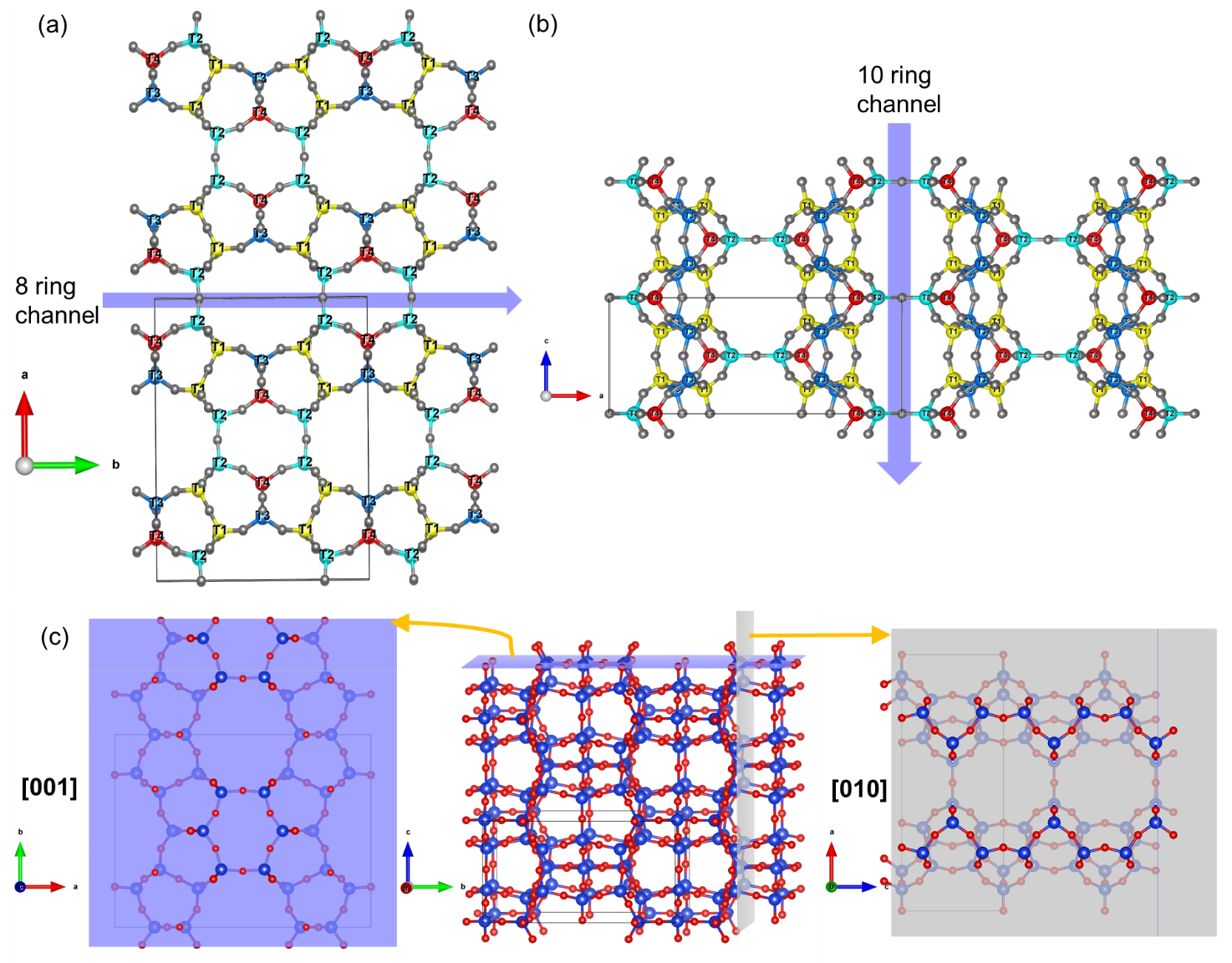


**Figure S5** Four T sites of FER zeolite view from (a) the crystallographic [001] direction, (b) the crystallographic [010] direction, and (c) the lattice planes of [001] (Purple) and [010] (grey).

**
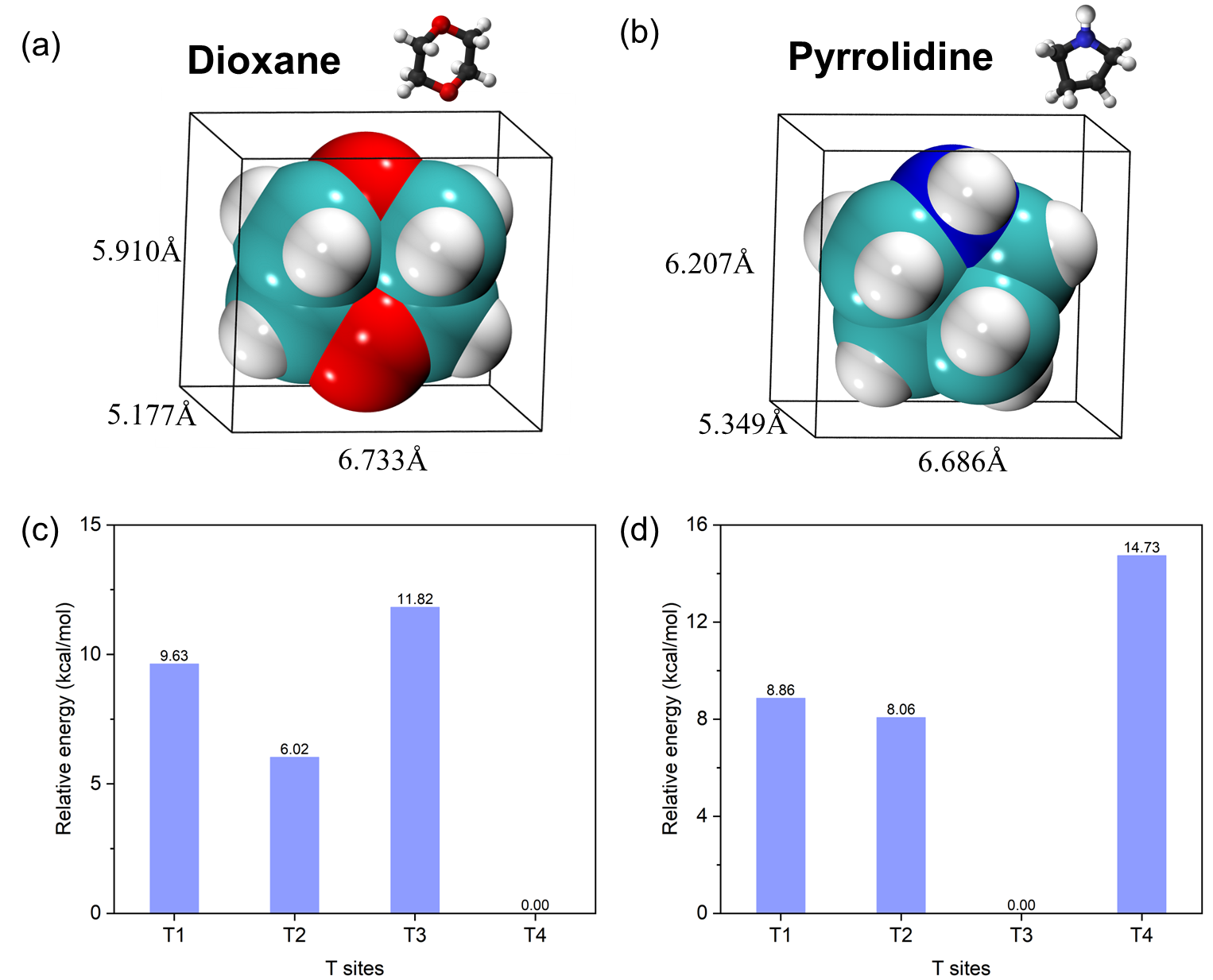
**

**Figure S6** The molecular size of (a) dioxane and (b) pyrrolidine. Relative energies of (c) dioxane and (d) protonated pyrrolidine trapped in the FER zeolite.


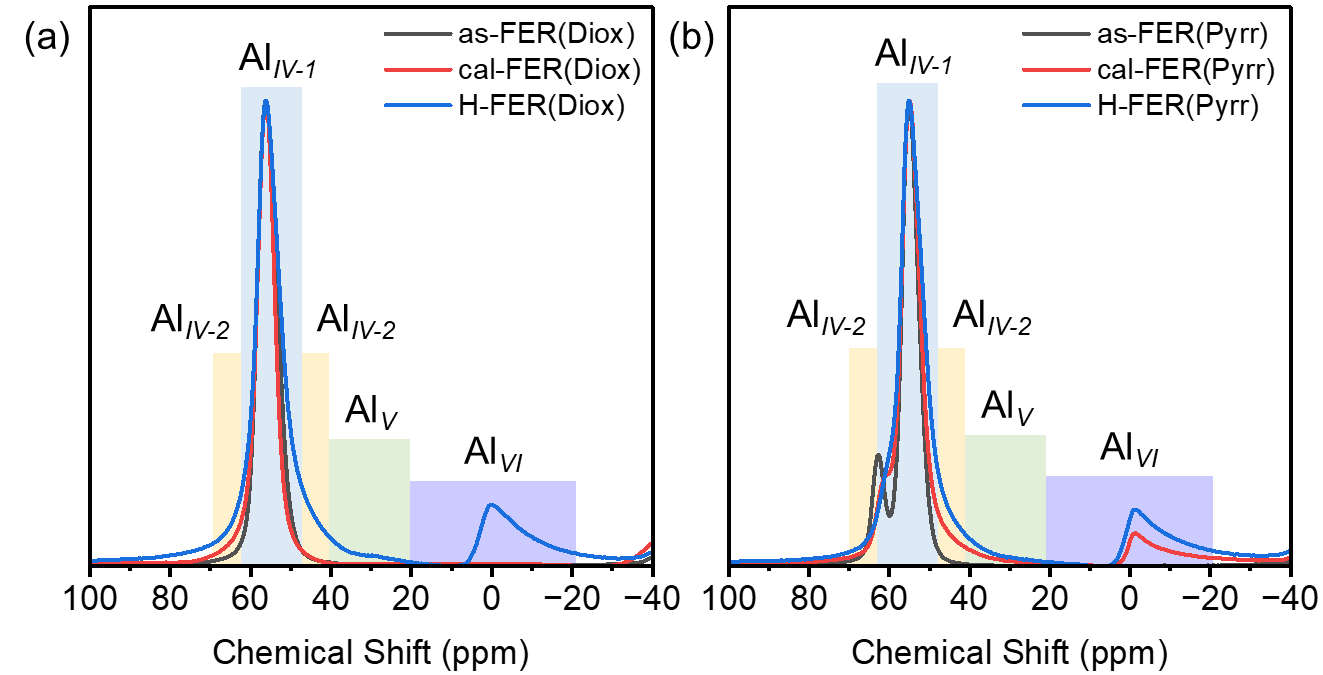


**Figure S7** ^27^Al MAS NMR spectra for (a) FER(Diox) and (b) FER(Pyrr).


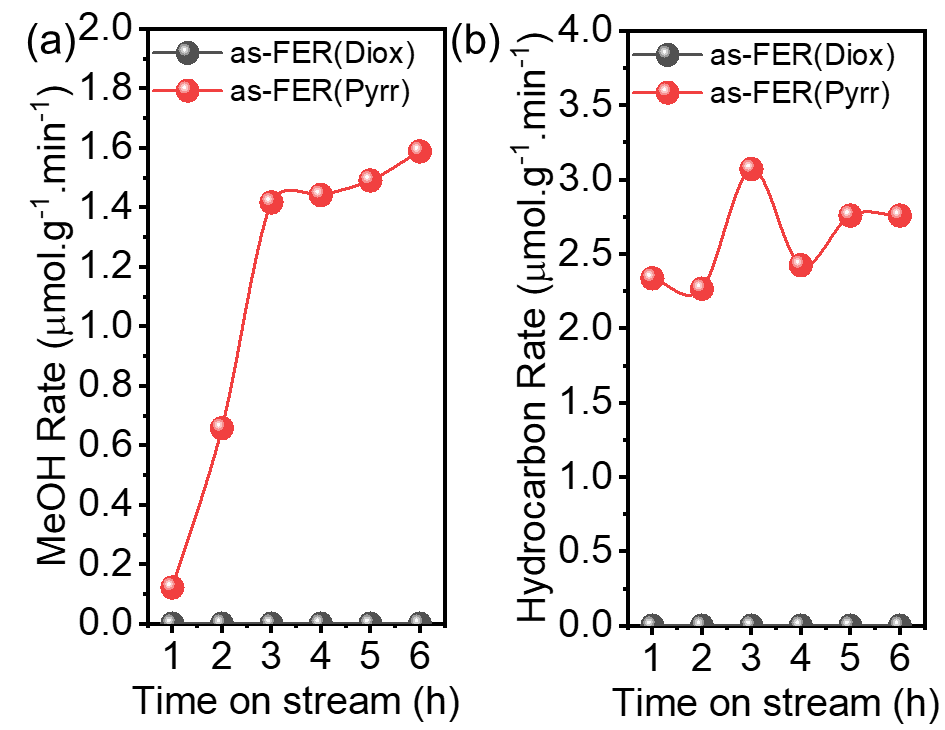


**Figure S8** Comparison of (a) methanol formation rate and (b) hydrocarbon formation rate of as-FER(Diox) and as-FER(Pyrr) zeolites at 350 ^o^C without activation.

**
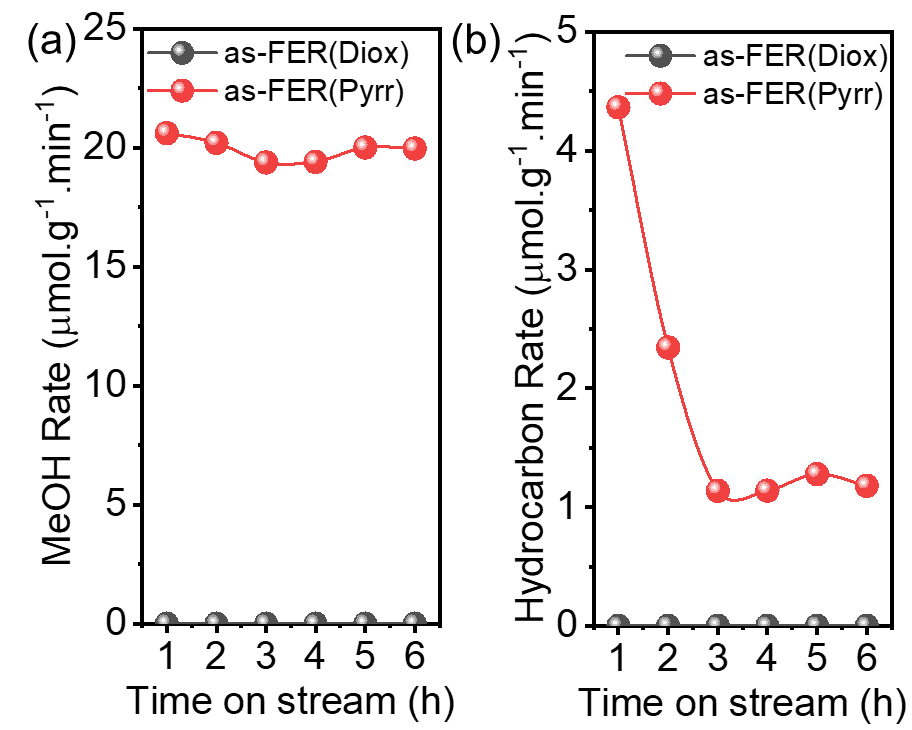
**

**Figure S9** Comparison of (a) methanol formation rate and (b) hydrocarbon formation rate of as-FER(Diox) and as-FER(Pyrr) zeolites at 350 ^o^C with activation at 500 ^o^C for 1 h.

**
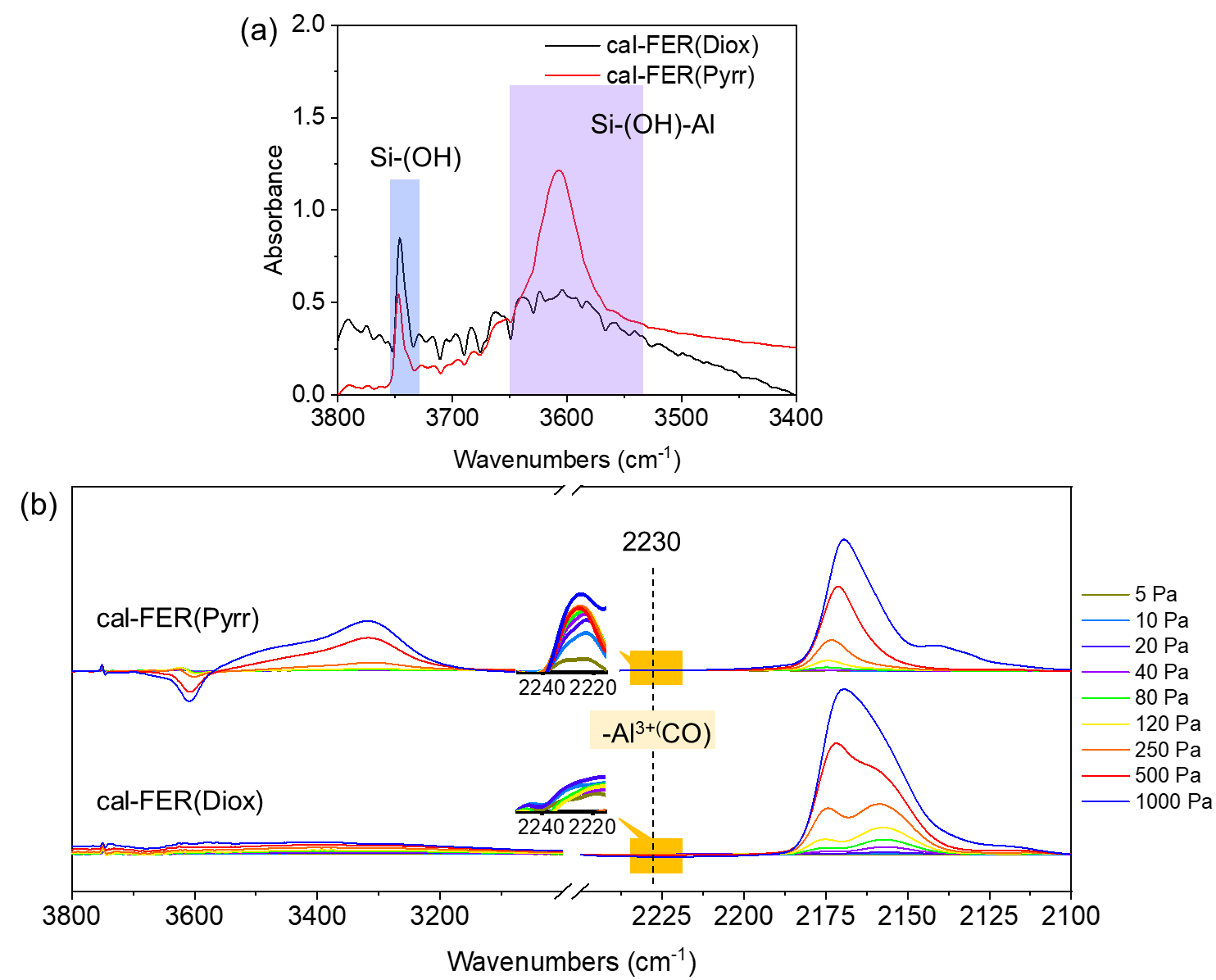
**

**Figure S10** (a) Hydroxy of cal-FER(Diox) and cal-FER(Pyrr), (b) CO adsorption FTIR spectra of cal-FER(Diox) and cal-FER(Pyrr) at -120 ^o^C after activation at 500 ^o^C for 1 h.


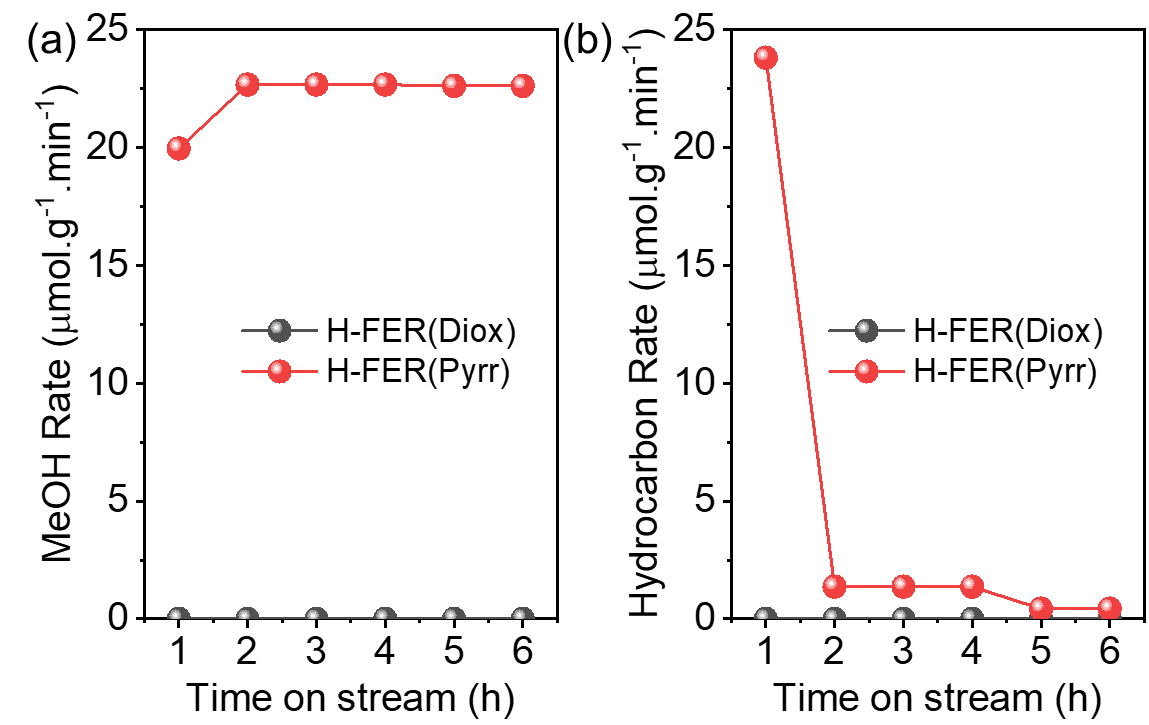


**Figure S11** Comparison of (a) methanol formation rate and (b) hydrocarbon formation rate of cal-FER(Diox) and cal-FER(Pyrr) zeolites at 350 ^o^C with activation at 500 ^o^C for 1 h.


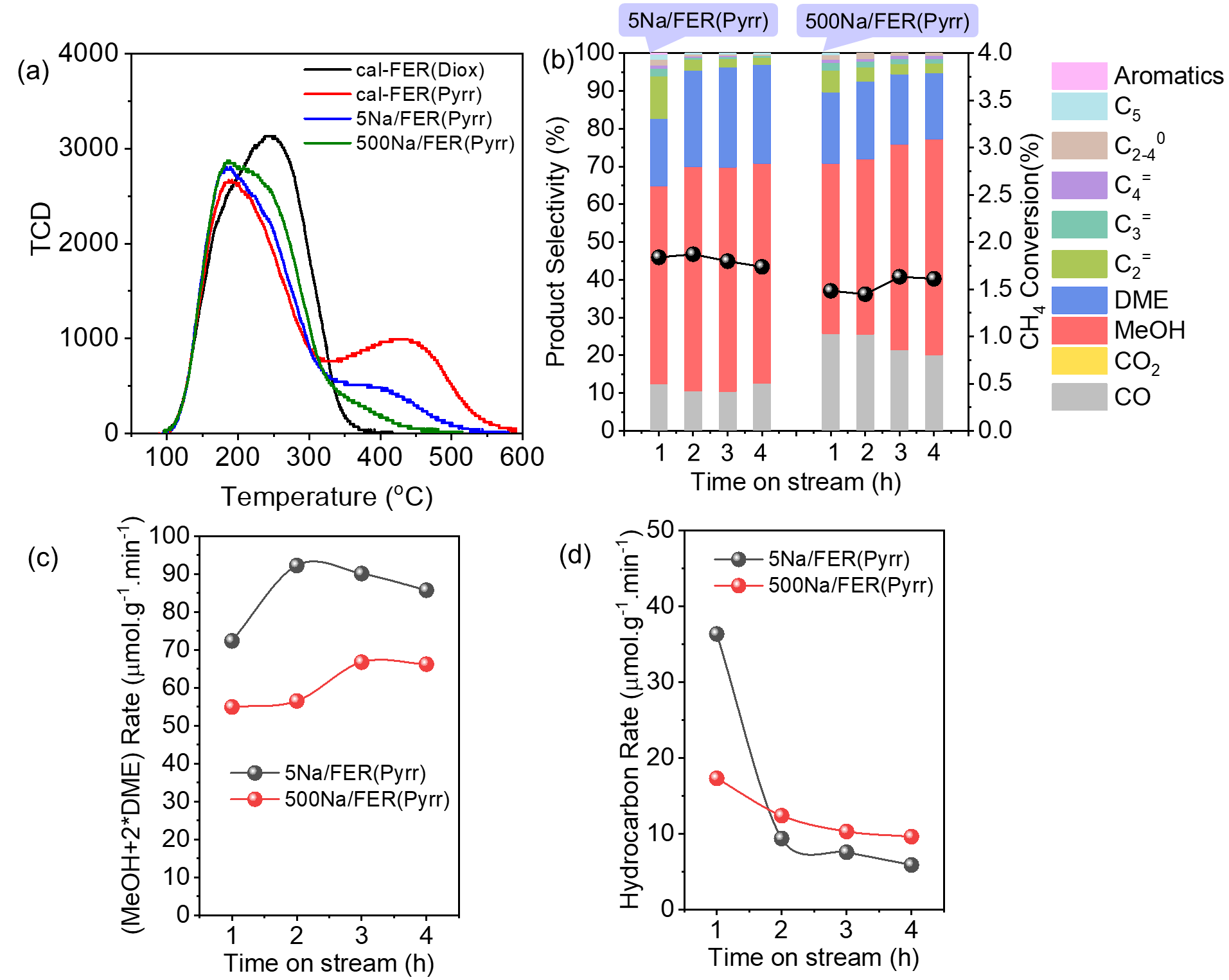


**Figure S12** Comparison of (a) NH_3_-TPD curves of FER zeolite, (b) product distribution and CH_4_ conversion of Na/FER zeolite, (c) methanol formation rate and (d) hydrocarbon formation rate of Na/FER(Pyrr) zeolites at 350 ^o^C.


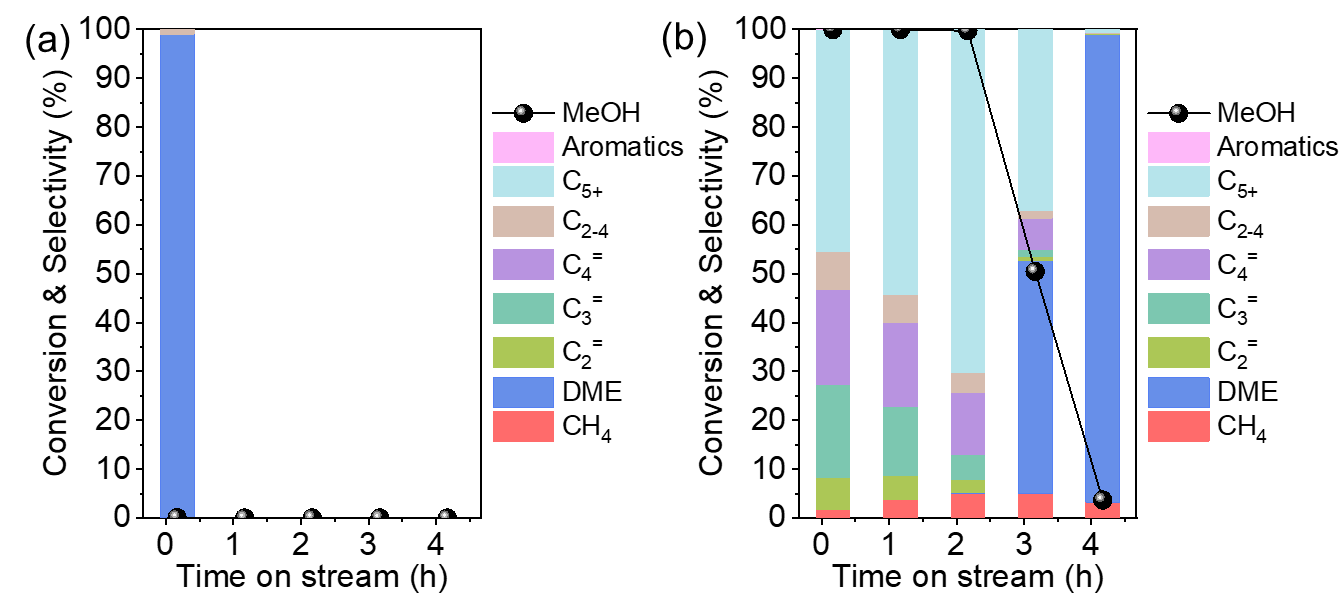


**Figure S13** Time courses the methane to hydrocarbon reaction at 350 °C on (a) cal-FER(Diox) and (b)cal-FER(Pyrr) zeolite catalyst. Reaction condition: 100 mg catalyst, 5 vol% methanol in Ar gas, W/F_MeOH_ = 68 g·h·mol^-1^.

**Figure S14** N_2_ adsorption and desorption isotherms of H-FER(Diox) and H-FER(Pyrr).


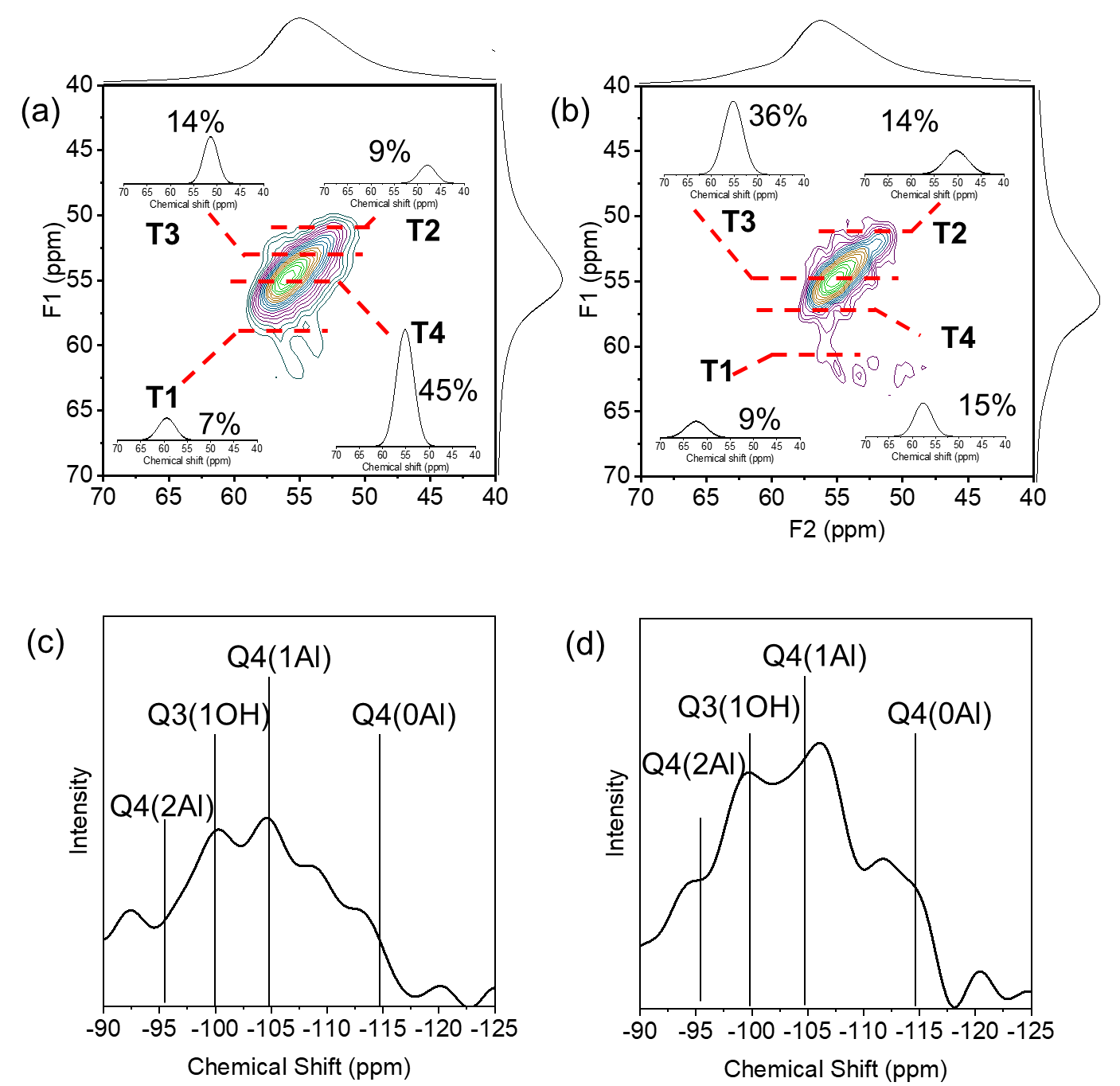


**Figure S15** ^27^Al MQMAS NMR spectra for (a) H-FER(Diox) and (b) H-FER(Pyrr).^29^Si CPMAS NMR spectra for (c) H-FER(Diox) and (d) H-FER(Pyrr).


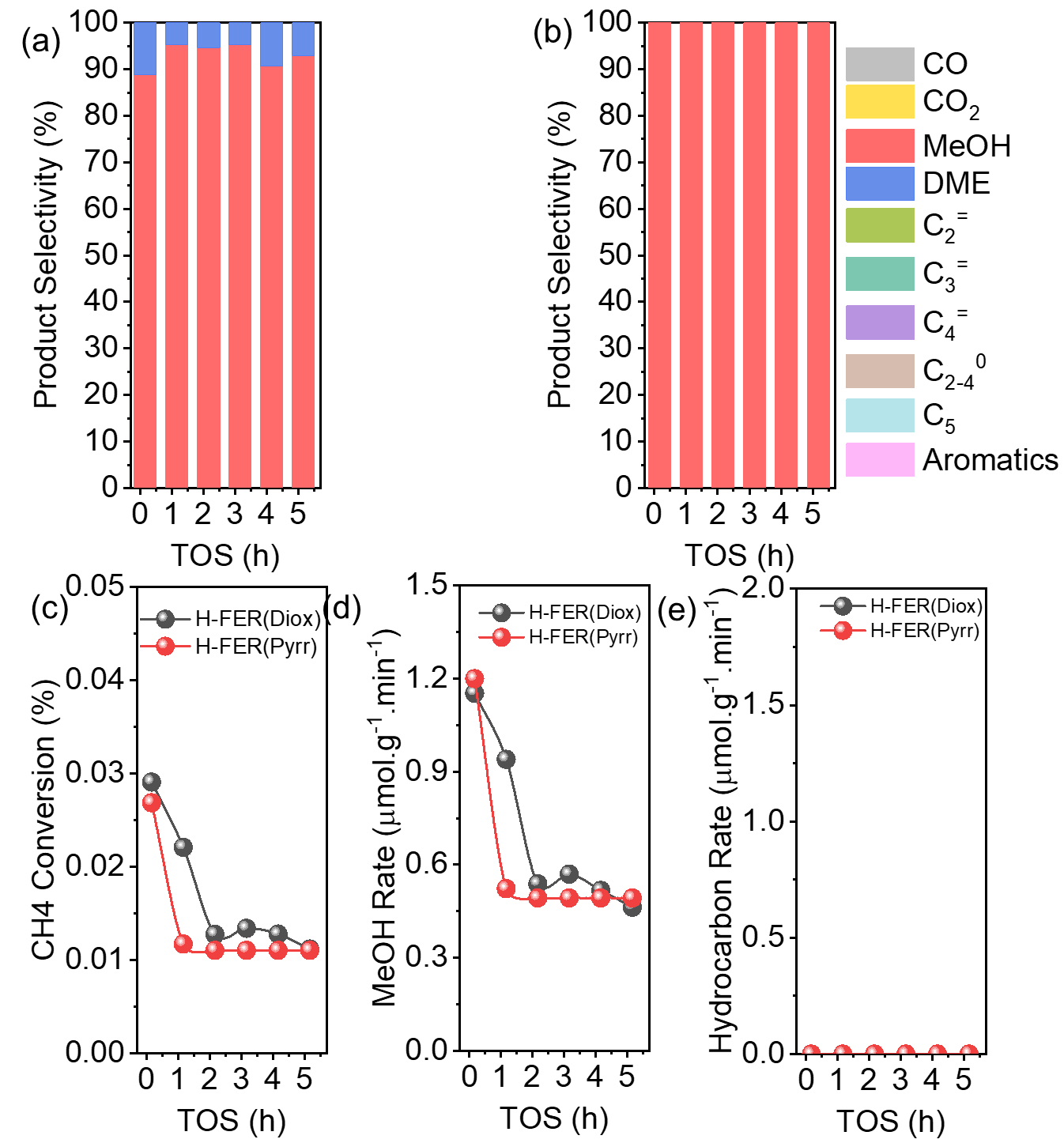


**Figure S16** Comparison of product distribution of (a) H-FER(Diox) and (b) H-FER(Pyrr) in the methane oxidation reaction, (c) CH_4_ conversion, (d) methanol formation rate, and (e) hydrocarbon formation rate of H-FER(Diox) and H-FER(Pyrr) zeolites at 250 ^o^C. Reaction conditions: 100 mg catalyst, CH_4_/N_2_O/H_2_O/Ar = 10/10/2/3 ml·min^-1^, WHSV =15000 ml·g^-1^·h^-1^.

**
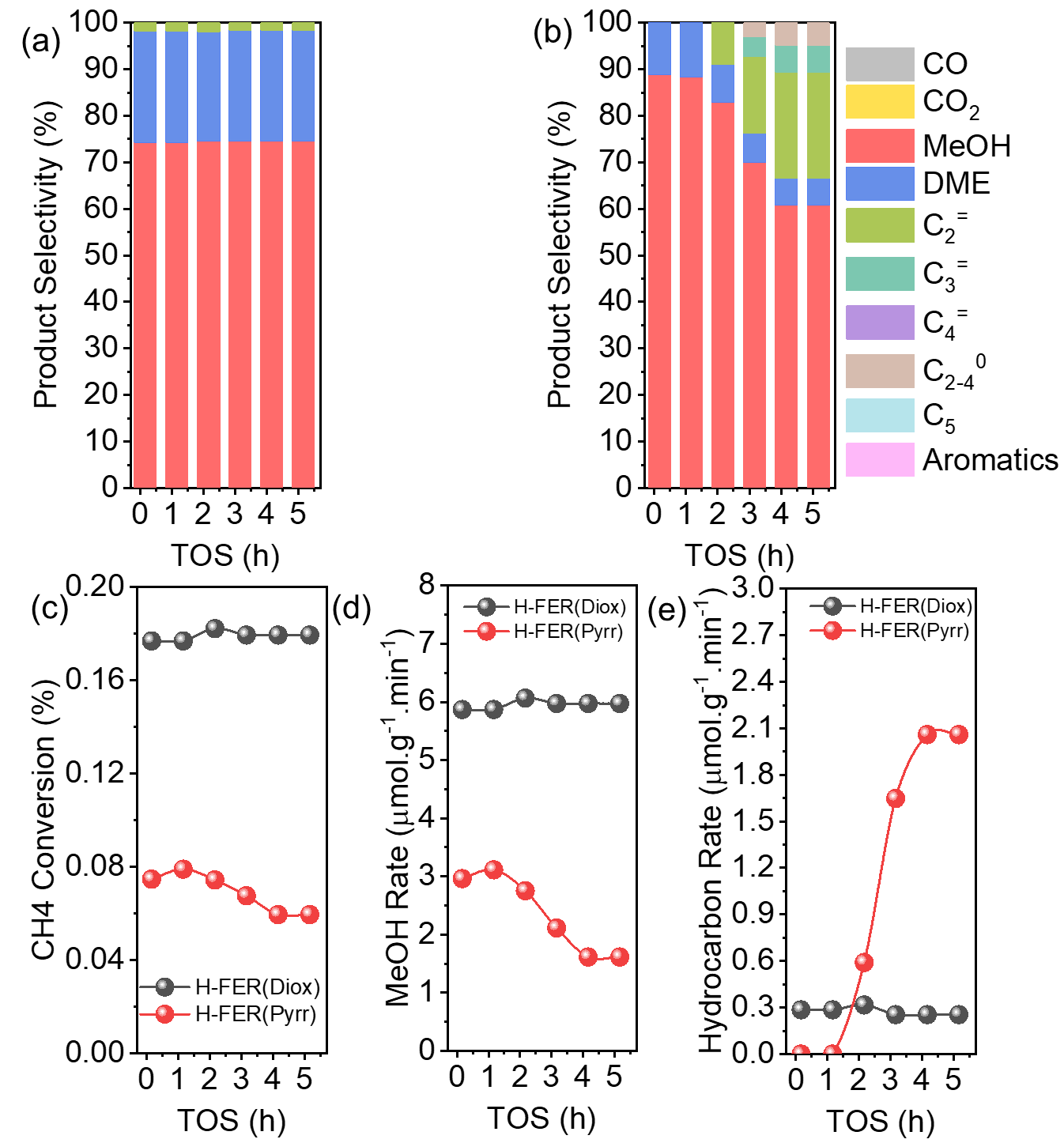
**

**Figure S17** Comparison of product distribution of (a) H-FER(Diox) and (b) H-FER(Pyrr) in the methane oxidation reaction, (c) CH_4_ conversion, (d) methanol formation rate, and (e) hydrocarbon formation rate of H-FER(Diox) and H-FER(Pyrr) zeolites at 275 ^o^C. Reaction conditions: 100 mg catalyst, CH_4_/N_2_O/H_2_O/Ar = 10/10/2/3 ml·min^-1^, WHSV =15000 ml·g^-1^·h^-1^.

**
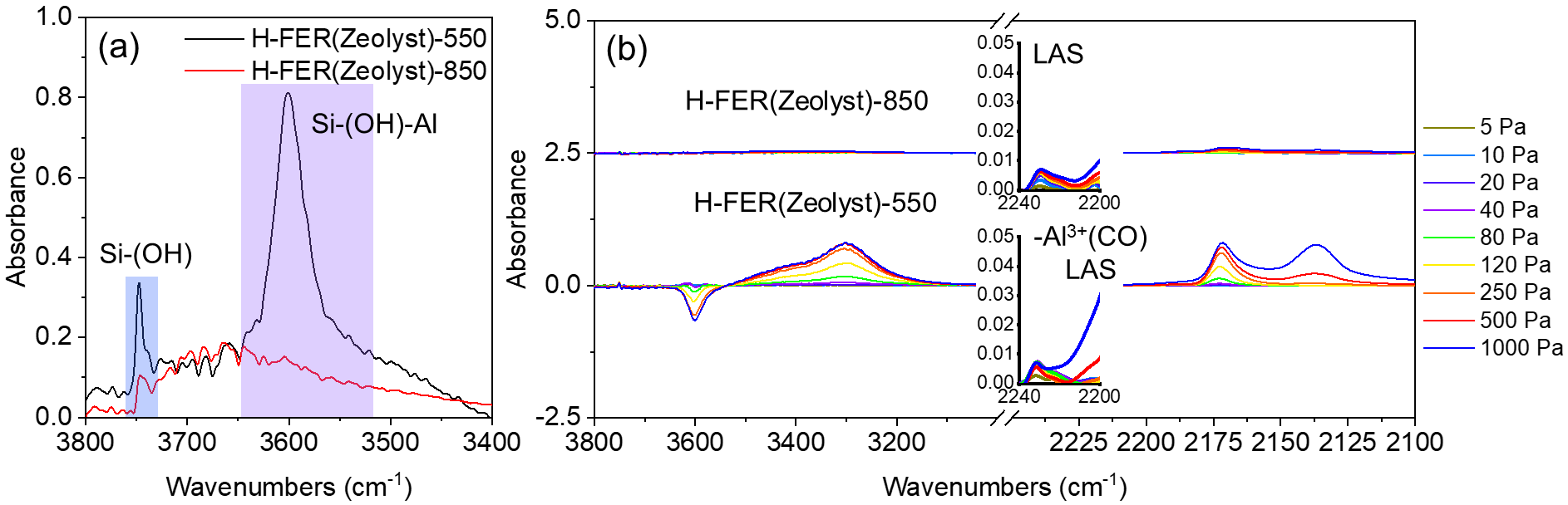
Figure S18** (a) Hydroxy of H-FER(Zeolyst)-550 and H-FER(Zeolyst)-850, (b) CO adsorption FTIR spectra of H-FER(Zeolyst)-550 and H-FER(Zeolyst)-850 at -120 ^o^C after activation at 500 ^o^C for 1 h.


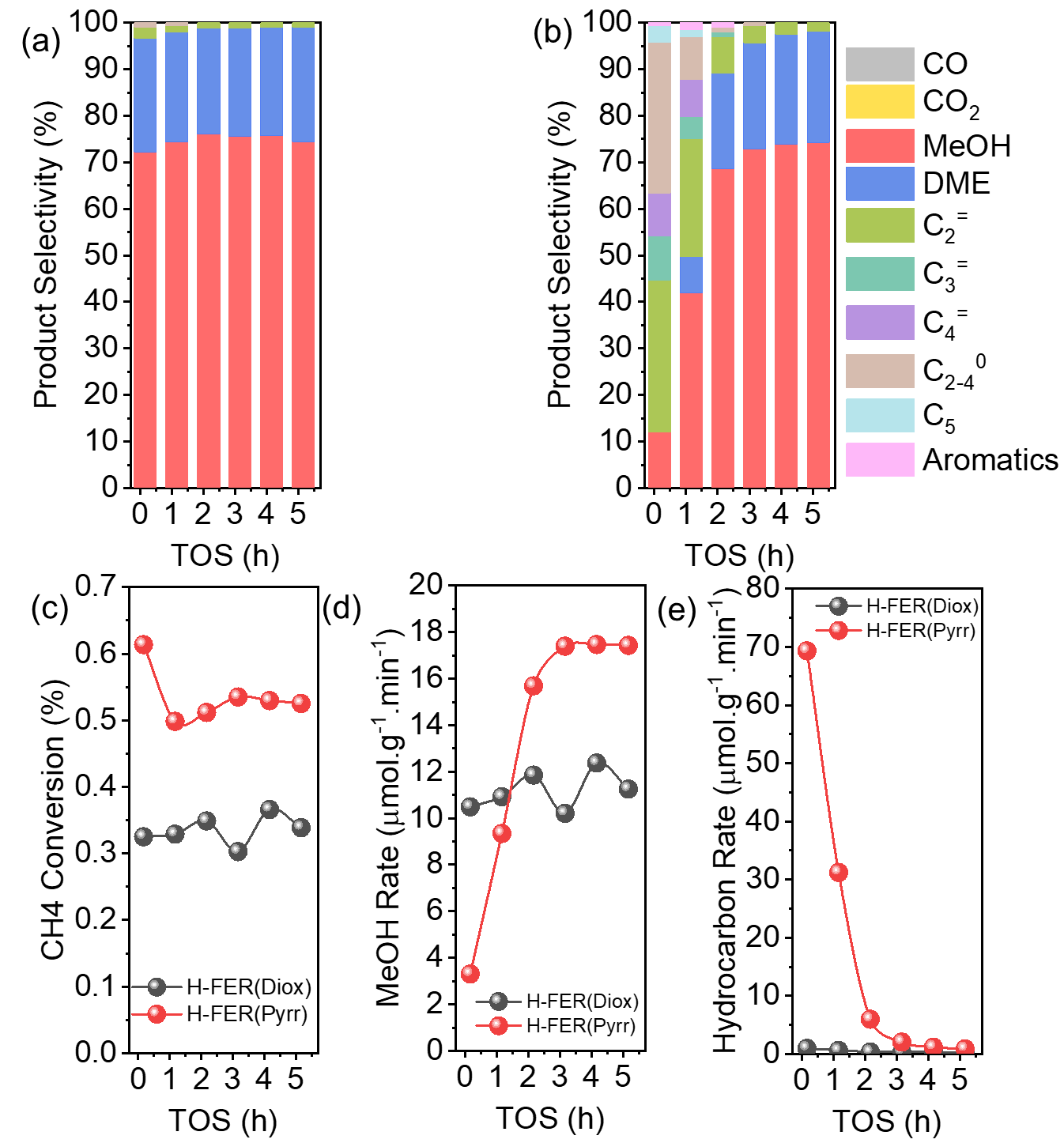


**Figure S19** Comparison of product distribution of (a) H-FER(Diox) and (b) H-FER(Pyrr) in the methane oxidation reaction, (c) CH_4_ conversion, (d) methanol formation rate, and (e) hydrocarbon formation rate of H-FER(Diox) and H-FER(Pyrr) zeolite at 300 ^o^C. Reaction conditions: 100 mg catalyst, CH_4_/N_2_O/H_2_O/Ar = 10/10/2/3 ml·min^-1^, WHSV =15000 ml·g^-1^·h^-1^.

**
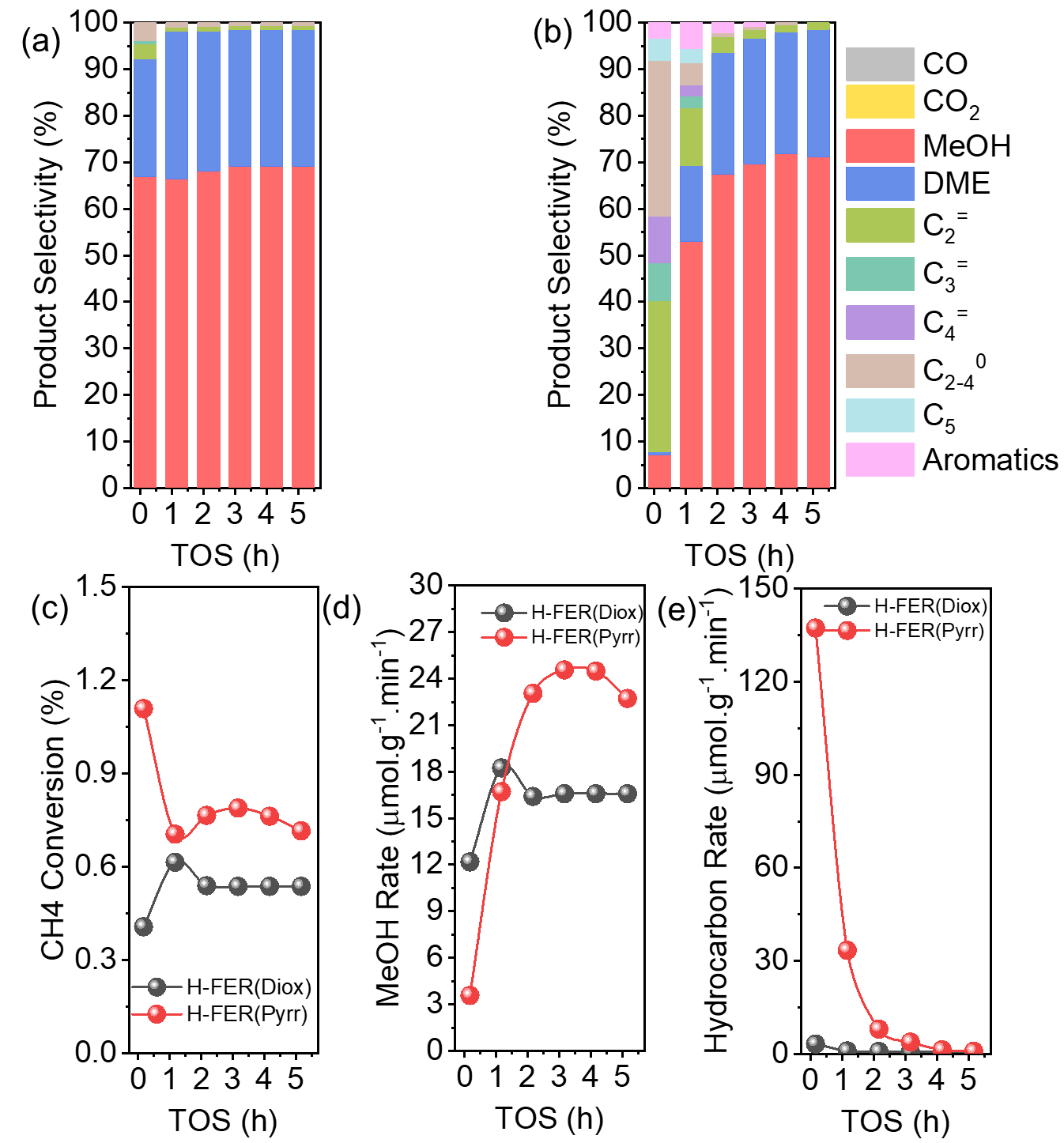
**

**Figure S20** Comparison of product distribution of (a) H-FER(Diox) and (b) H-FER(Pyrr) in the methane oxidation reaction, (c) CH_4_ conversion, (d) methanol formation rate, and (e) hydrocarbon formation rate of H-FER(Diox) and H-FER(Pyrr) zeolites at 325 ^o^C. Reaction conditions: 100 mg catalyst, CH_4_/N_2_O/H_2_O/Ar = 10/10/2/3 ml·min^-1^, WHSV =15000 ml·g^-1^·h^-1^.


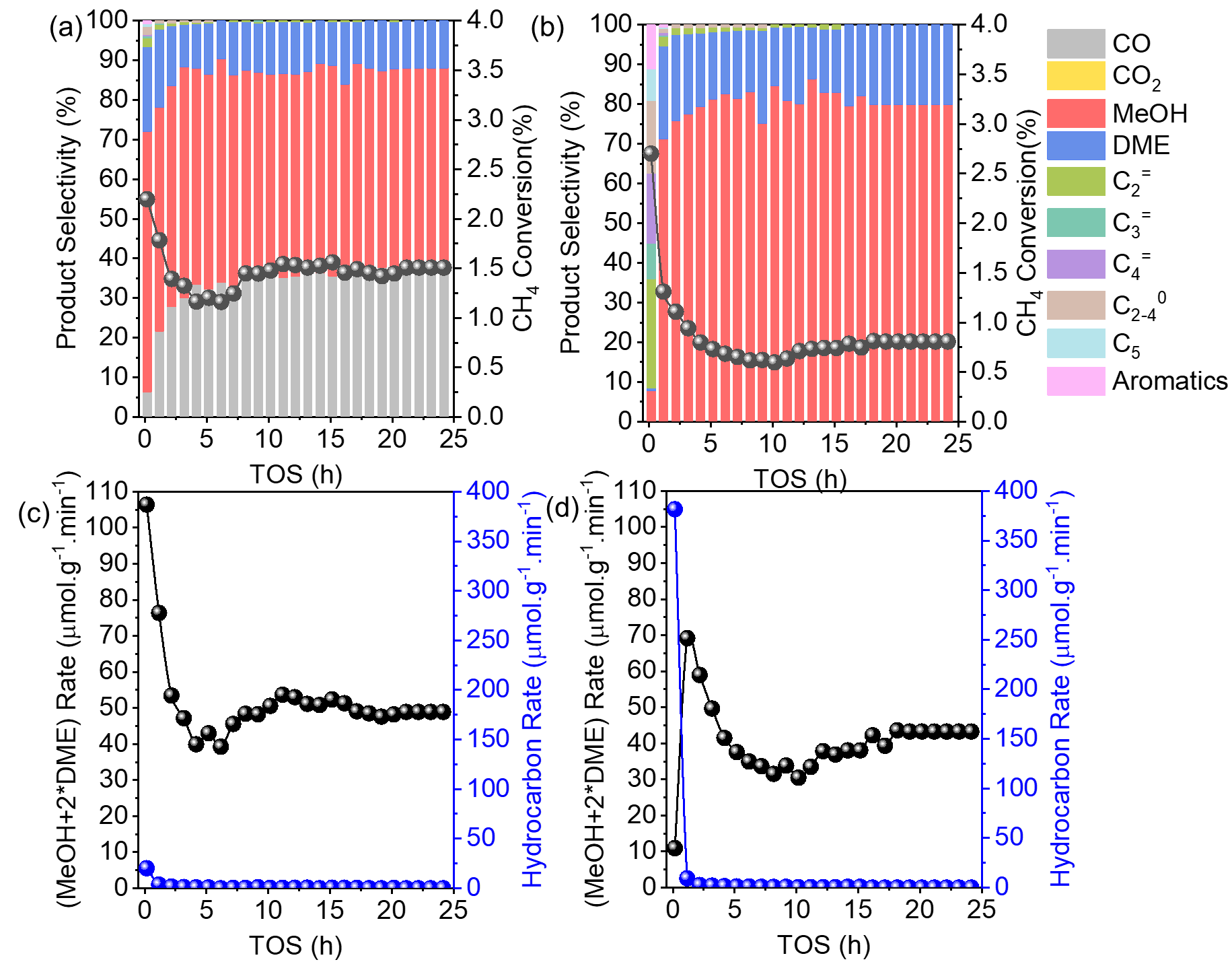


**Figure S21** Product distribution and CH_4_ conversion of (a) H-FER(Diox) and (b) H-FER(Pyrr) in the methane oxidation reaction. (MeOH+2*DME) formation rate and hydrocarbon formation rate of (c) H-FER(Diox) and (d) H-FER(Pyrr) zeolites at 350 ^o^C. Reaction conditions: 100 mg catalyst, CH_4_/N_2_O/H_2_O/Ar = 10/10/2/3 ml·min^-1^, WHSV =15000 ml·g^-1^·h^-1^.


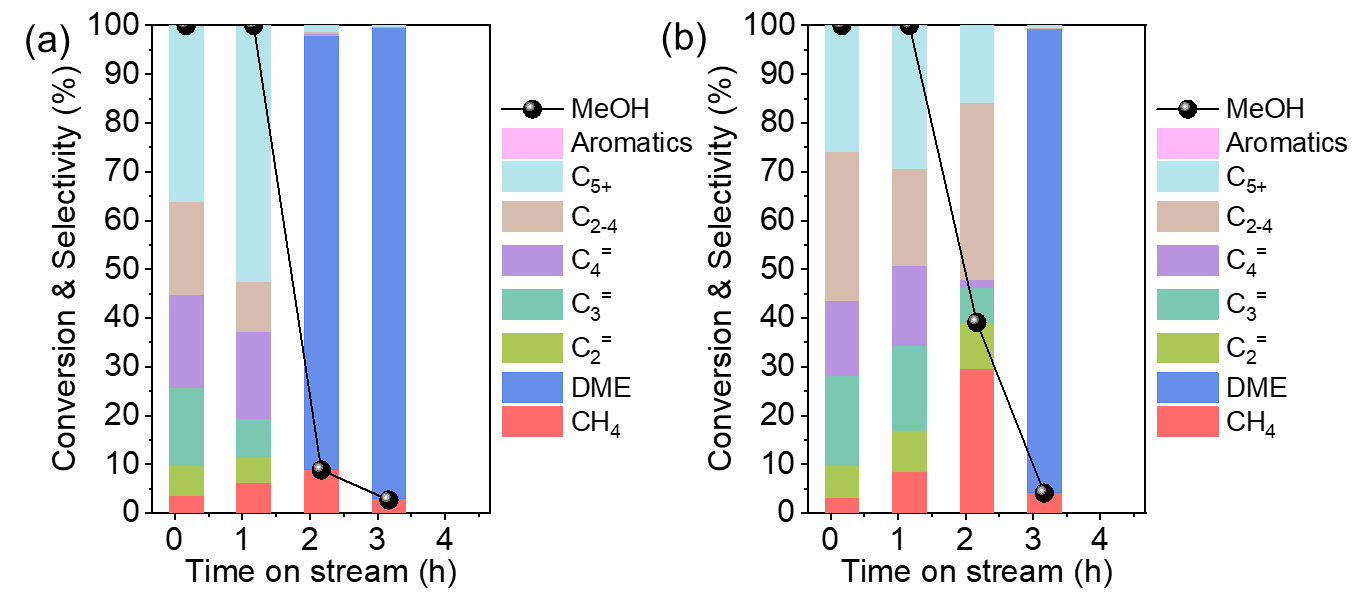
**Figure S22** Time courses the methane to hydrocarbon reaction at 350 °C on (a) H-FER(Diox) and (b) H-FER(Pyrr) zeolite catalyst. Reaction condition: 100 mg catalyst, 5 vol% methanol in Ar gas, W/F_MeOH_ = 68 g·h·mol^-1^.


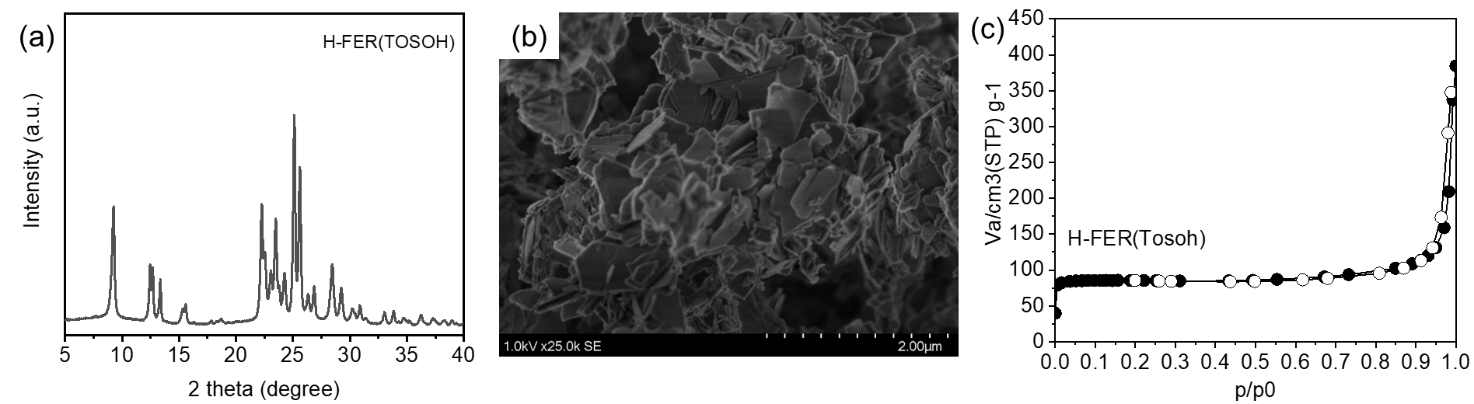


**Fig. S23** (a) XRD pattern, (b) SEM image, and (c) N_2_ adsorption and desorption isothermal of FER(Tosoh) zeolite.


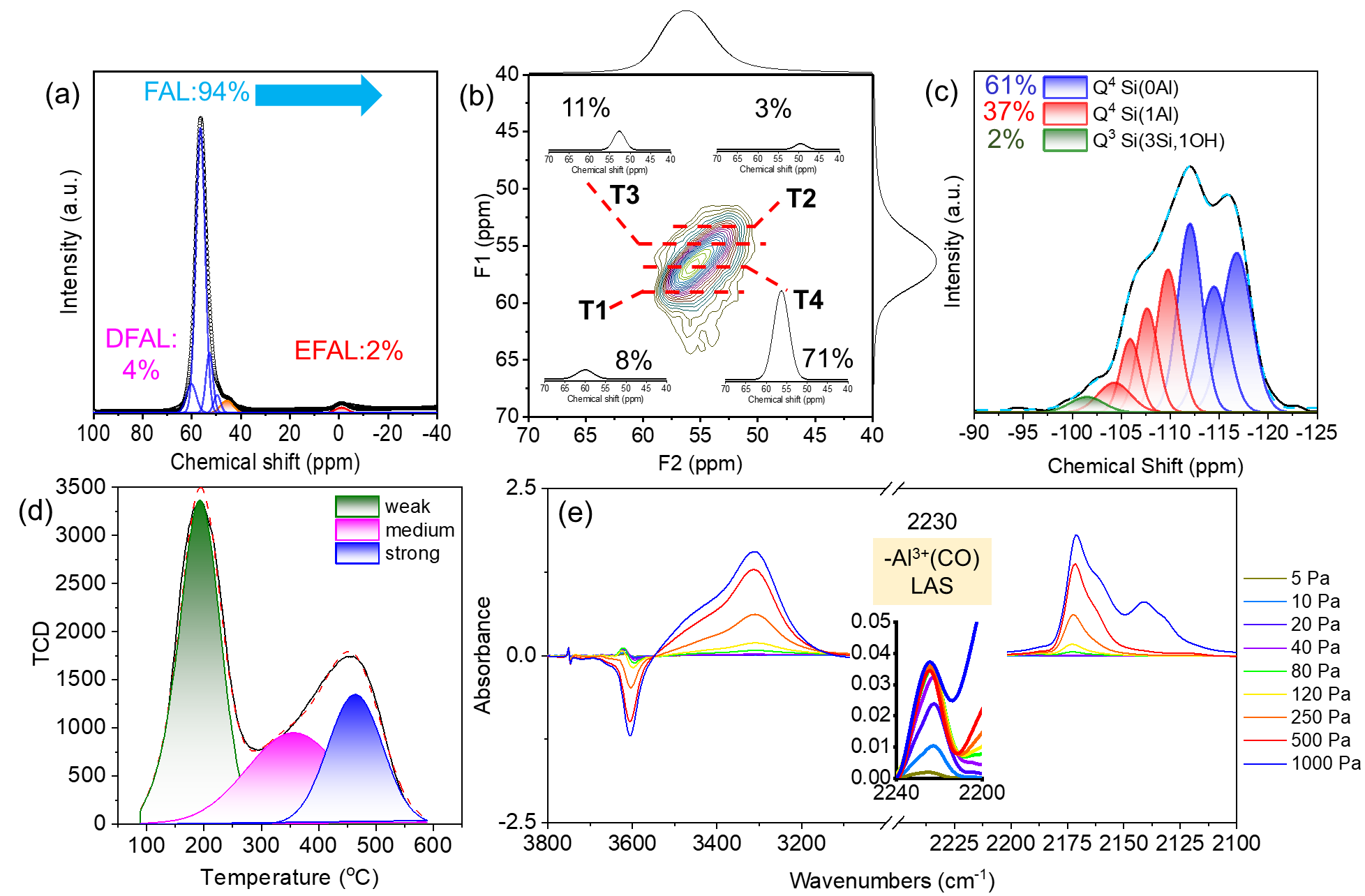


**Figure S24** (a) ^27^Al MAS NMR spectrum, (b) ^27^Al MQMAS NMR spectrum, (c) ^29^Si MAS NMR spectrum, (d) NH_3_-TPD curve, (e) CO adsorption FTIR spectra of H-FER(Tosoh) at -120 ^o^C after activation at 500 ^o^C for 1 h.


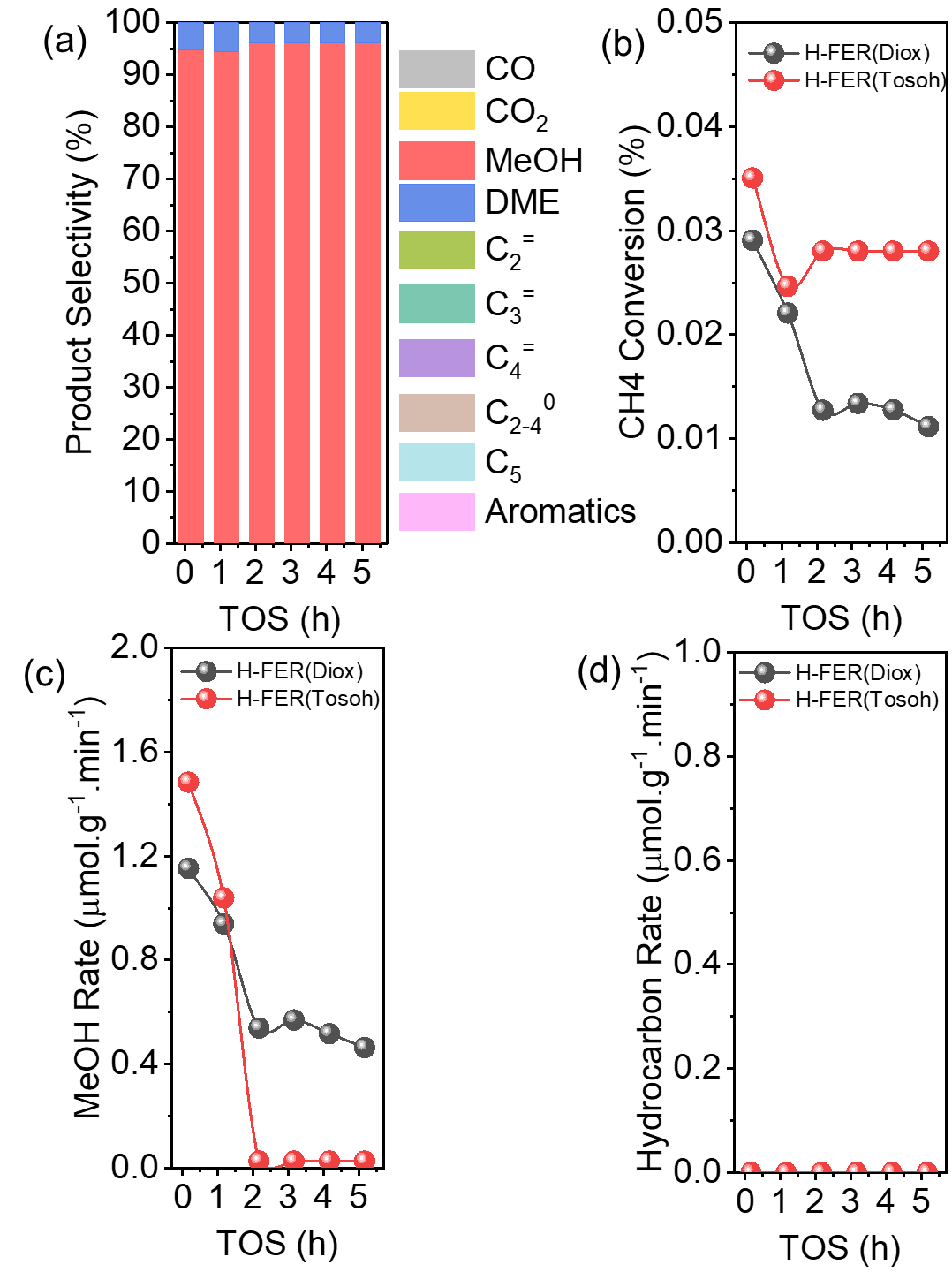


**Figure S25** (a) Product distribution of H-FER(Tosoh) in the methane oxidation reaction, comparison of (b) CH_4_ conversion, (c) methanol formation rate, and (d) hydrocarbon formation rate of H-FER(Diox) and H-FER(Tosoh) zeolites at 250 ^o^C. Reaction conditions: 100 mg catalyst, CH_4_/N_2_O/H_2_O/Ar = 10/10/2/3 ml·min^-1^, WHSV =15000 ml·g^-1^·h^-1^.

**
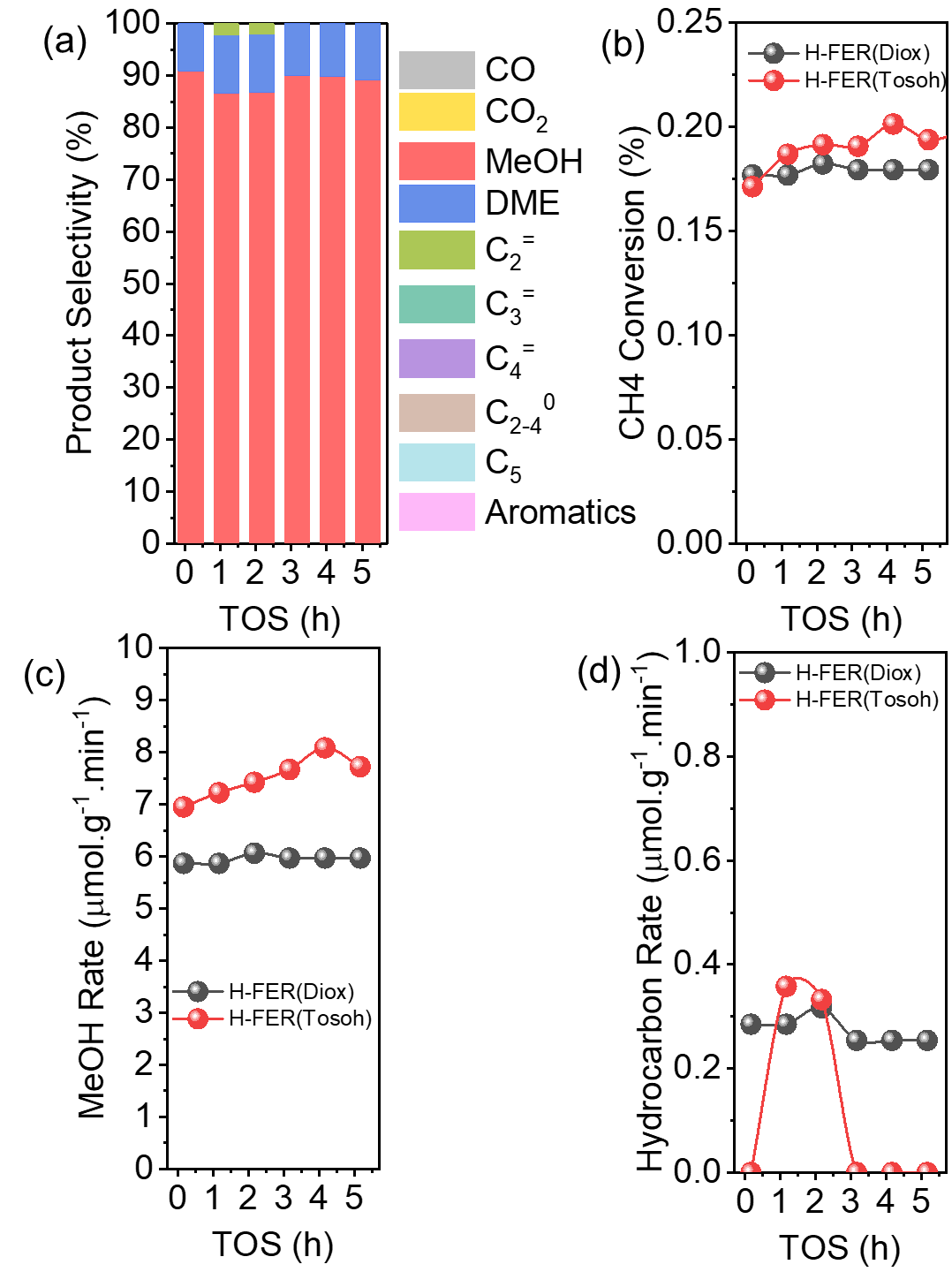
**

**Figure S26** (a) Product distribution of H-FER(Tosoh) in the methane oxidation reaction, comparison of (b) CH_4_ conversion, (c) methanol formation rate, and (d) hydrocarbon formation rate of H-FER(Diox) and H-FER(Tosoh) zeolites at 275 ^o^C. Reaction conditions: 100 mg catalyst, CH_4_/N_2_O/H_2_O/Ar = 10/10/2/3 ml·min^-1^, WHSV =15000 ml·g^-1^·h^-1^.

**
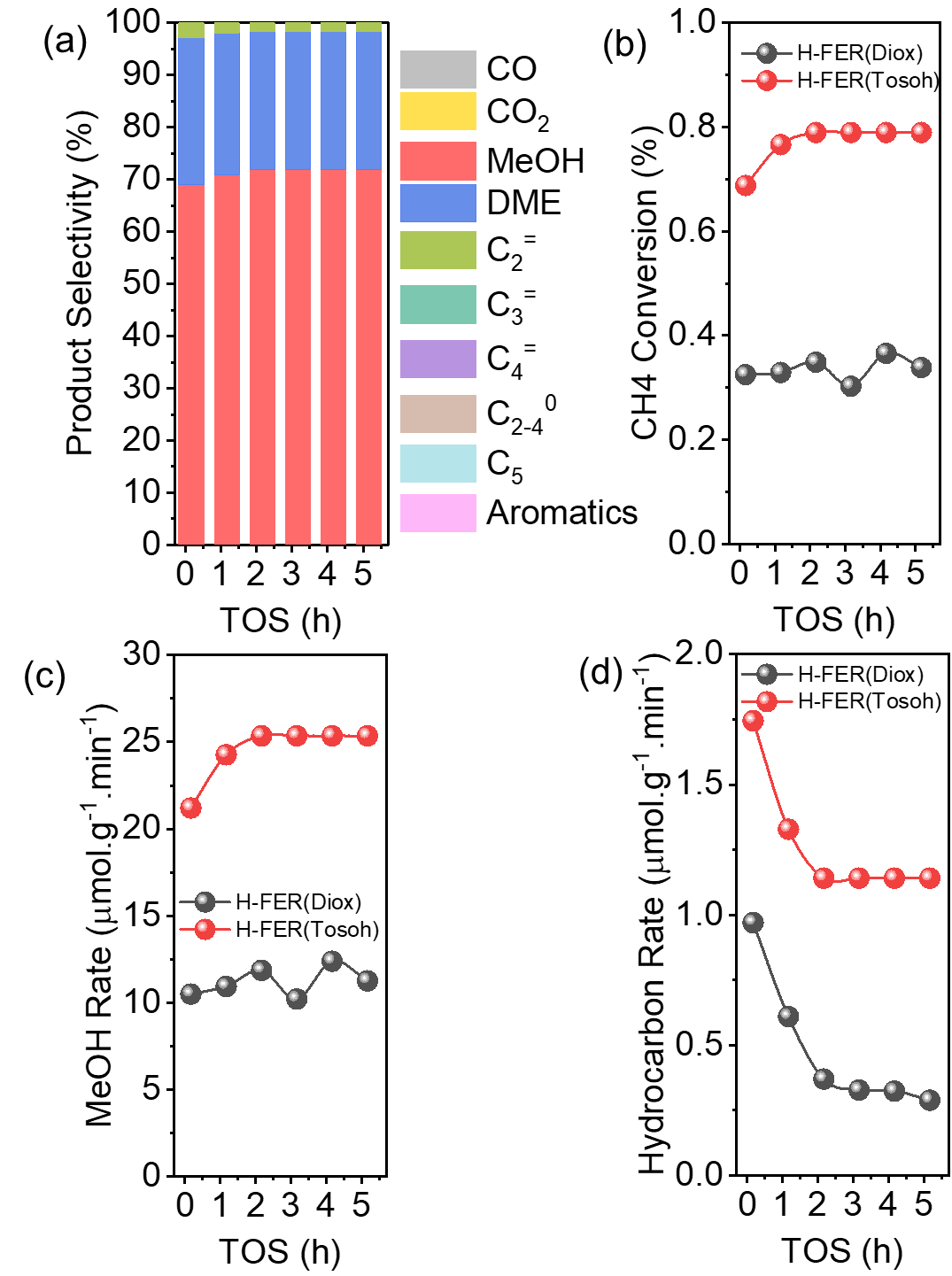
**

**Figure S27** (a) Product distribution of H-FER(Tosoh) in the methane oxidation reaction, comparison of (b) CH_4_ conversion, (c) methanol formation rate, and (d) hydrocarbon formation rate of H-FER(Diox) and H-FER(Tosoh) zeolites at 300 ^o^C. Reaction conditions: 100 mg catalyst, CH_4_/N_2_O/H_2_O/Ar = 10/10/2/3 ml·min^-1^, WHSV =15000 ml·g^-1^·h^-1^.

**
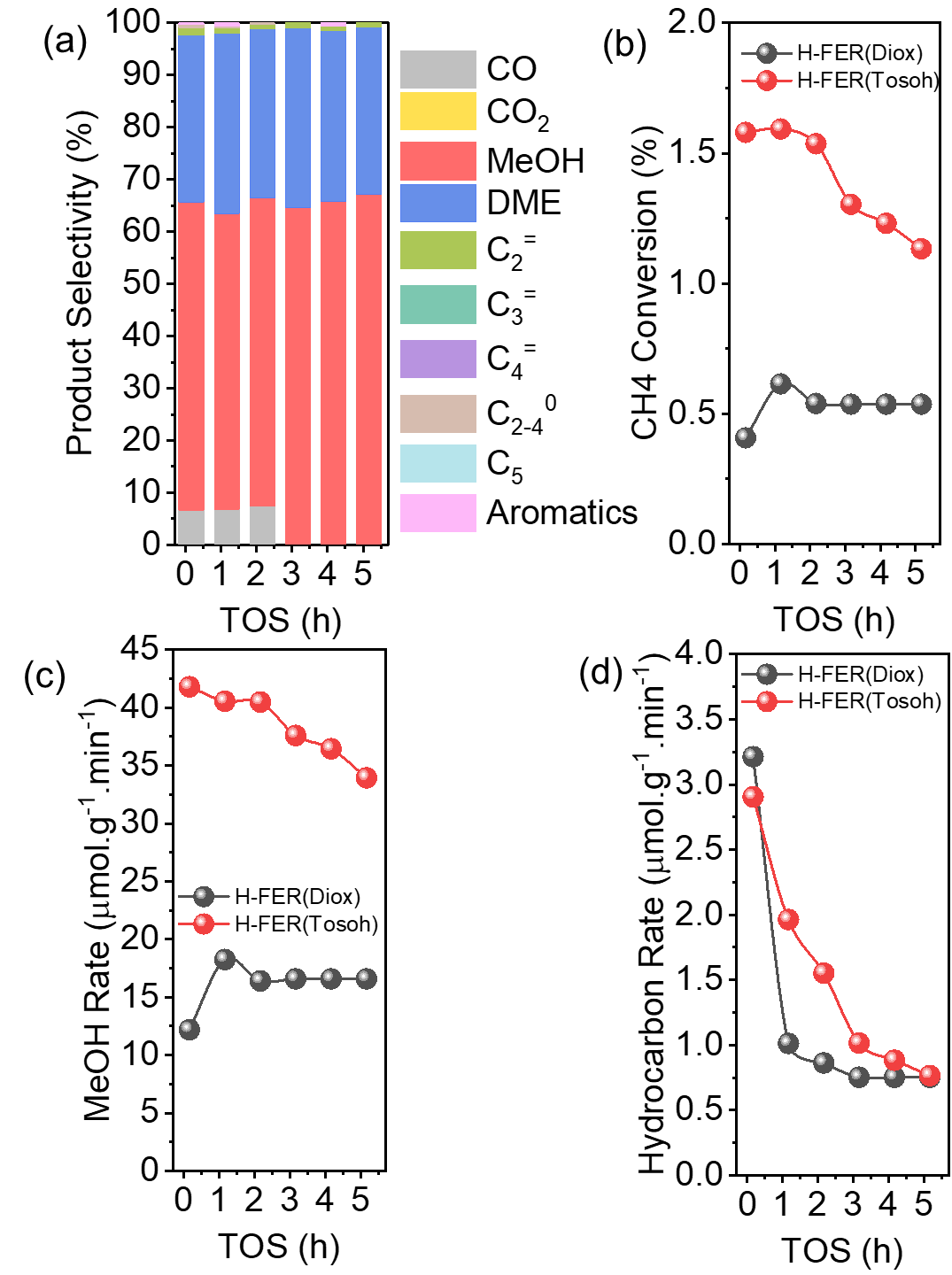
**

**Figure S28** (a) Product distribution of H-FER(Tosoh) in the methane oxidation reaction, comparison of (b) CH_4_ conversion, (c) methanol formation rate, and (d) hydrocarbon formation rate of H-FER(Diox) and H-FER(Tosoh) zeolite at 325 ^o^C. Reaction conditions: 100 mg catalyst, CH_4_/N_2_O/H_2_O/Ar = 10/10/2/3 ml·min^-1^, WHSV =15000 ml·g^-1^·h^-1^.

**
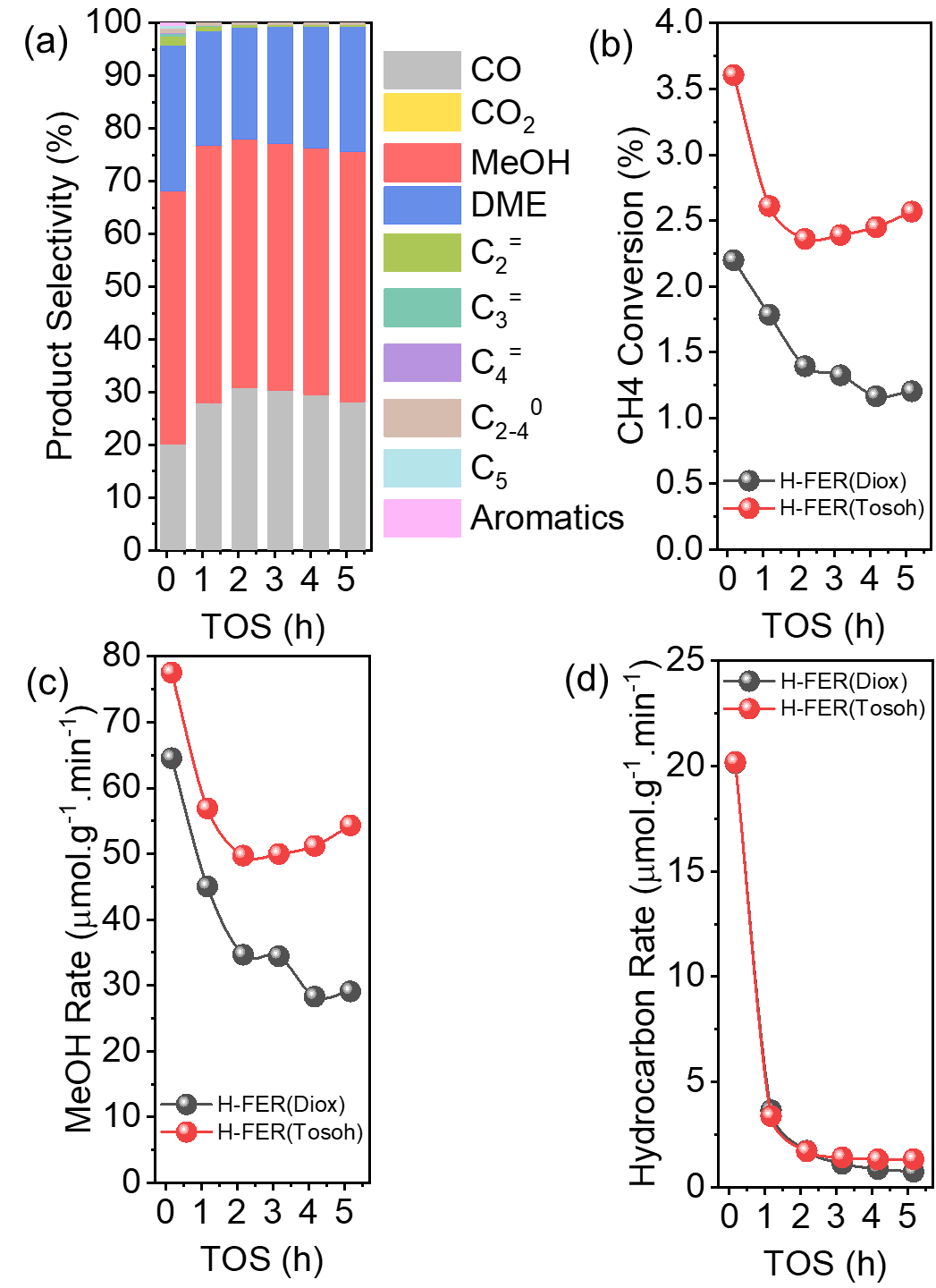
**

**Figure S29** (a) Product distribution of H-FER(Tosoh) in the methane oxidation reaction, comparison of (b) CH_4_ conversion, (c) methanol formation rate, and (d) hydrocarbon formation rate of H-FER(Diox) and H-FER(Tosoh) zeolites at 350 ^o^C. Reaction conditions: 100 mg catalyst, CH_4_/N_2_O/H_2_O/Ar = 10/10/2/3 ml·min^-1^, WHSV =15000 ml·g^-1^·h^-1^.

**
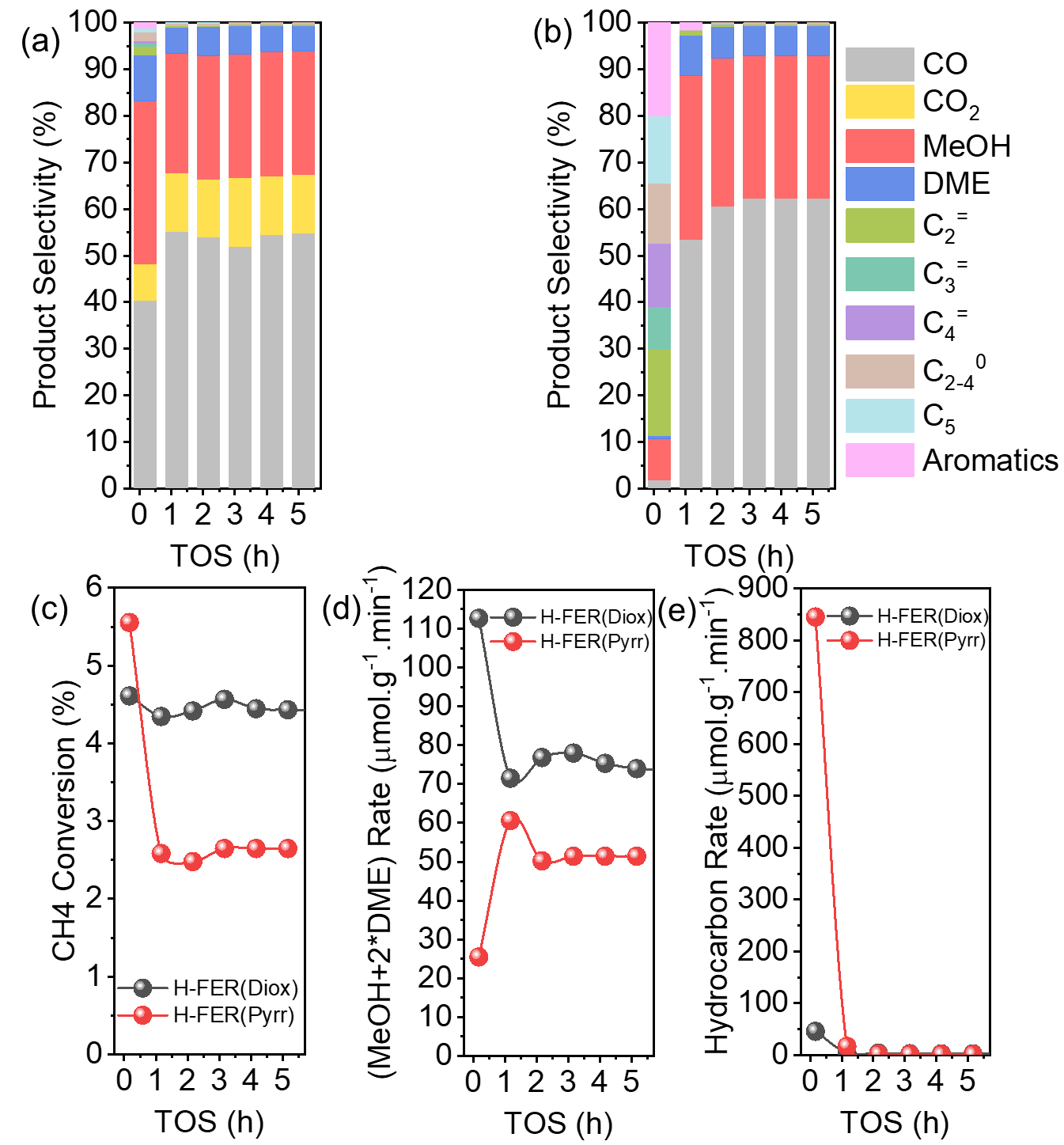
**

**Figure S30** Compare product distribution of (a) H-FER(Diox) and (b) H-FER(Pyrr) in the methane oxidation reaction, (c) CH_4_ conversion, (d) (MeOH+2*DME) formation rate, and (e) hydrocarbon formation rate of H-FER(Diox) and H-FER(Pyrr) zeolites at 375 ^o^C. Reaction conditions: 100 mg catalyst, CH_4_/N_2_O/H_2_O/Ar = 10/10/2/3 ml·min^-1^, WHSV =15000 ml·g^-1^·h^-1^.

**
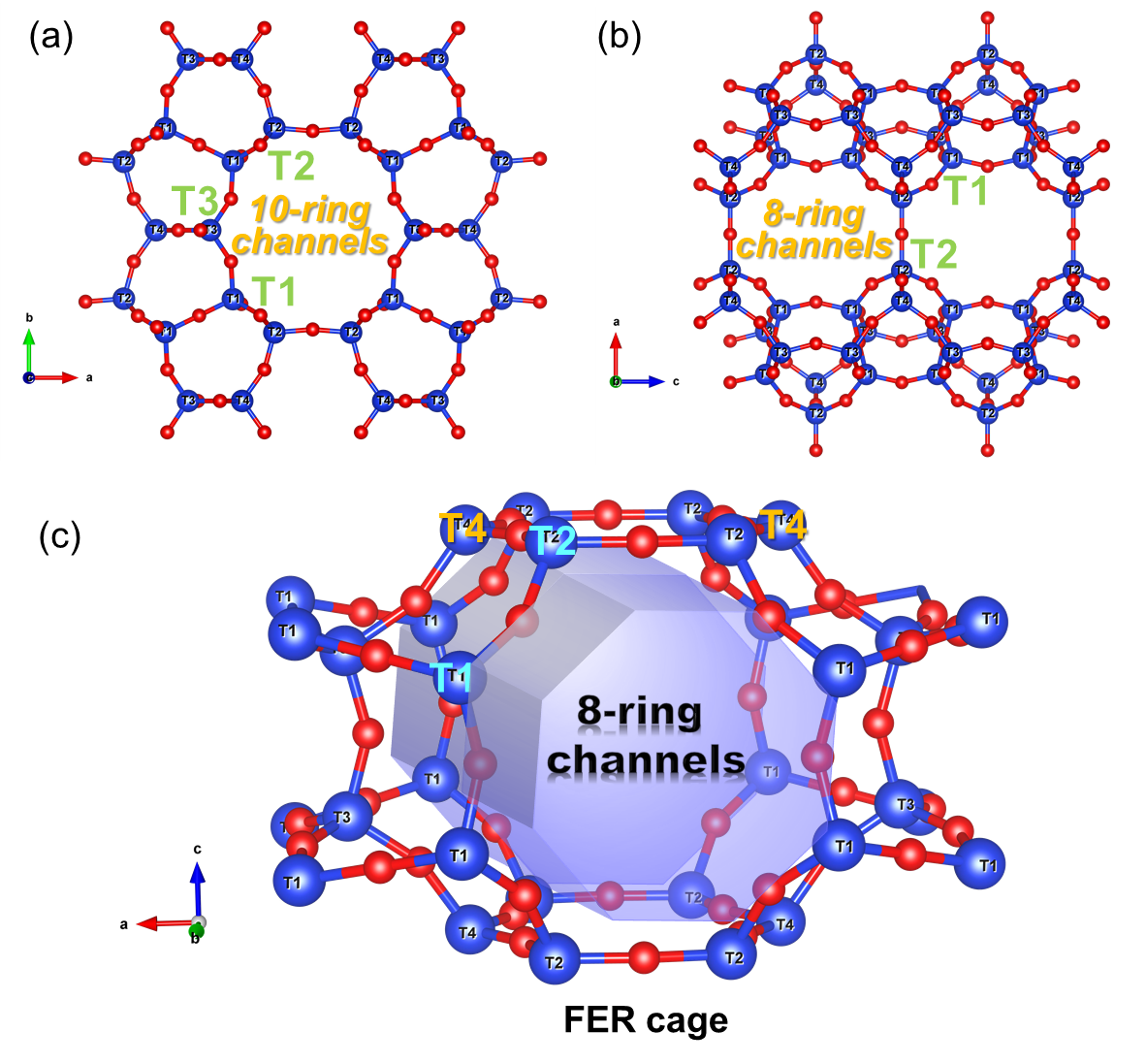
**

**Scheme S1.** (a) 10-ring channels, (b) 8-ring channels, and (c) FER cage with the corresponding T sites.

Table S1. Chemical composition and acid amount of FER zeolites.

| Sample | Chemical Compositions | | | Element / Unit cell | | | |
| --- | --- | --- | --- | --- | --- | --- | --- |
|  | Si/Al ^[a]^ | Na/Si ^[b]^ | OSDA/Si ^[c]^ | Al^[d]^ | OSDA^[e]^ | Na^[f]^ | H^[g]^ |
| as-FER(Diox) | 10.0 | 0.12 | 0.05 | 3.27 | 1.64 | 3.93 | 0 |
| cal-FER(Diox) | 10.0 | 0.12 | - | 3.27 | 0 | 3.93 | 0 |
| H-FER(Diox) | 10.0 | - | - | 3.27 | 0 | 0 | 3.27 |
| as-FER(Pyrr) | 9.3 | 0.03 | 0.09 | 3.50 | 2.93 | 0.93 | 0 |
| cal-FER(Pyrr) | 9.3 | 0.03 | - | 3.50 | 0 | 0.93 | 2.93 |
| H-FER(Pyrr) | 9.3 | - | - | 3.50 | 0 | 0 | 3.50 |

[a] by ICP-AES.

[b] by AAS.

[c] by TG-DTA, dioxane was a pore-filling agent.

[d] by Si/Al and the chemical formula of FER (|Mg_2_Na_2_(H_2_O)_18_ | [Al_6_Si_30_O_72_]-FER).

[e] by OSDA/Si and the chemical formula of FER.

[f] by Na/Al and the chemical formula of FER.

[g] by OSDA/Si and the chemical formula of FER, removed OSDA was replaced by H^+^.

Table S2. Textual properties of FER zeolites.

| Sample | Textual Properties | | | |
| --- | --- | --- | --- | --- |
|  | S_BET_(m^2^·g^-1^) ^a^ | V_Total_(cm^3^·g^-1^) ^a^ | S_EXT_(m^2^·g^-1^) ^b^ | V_Mic_(cm^3^·g^-1^) ^b^ |
| H-FER(Diox) | 299 | 0.31 | 35 | 0.11 |
| H-FER(Pyrr) | 311 | 0.34 | 39 | 0.12 |
| H-FER(Tosoh) | 310 | 0.47 | 58 | 0.10 |

*^a^* by the Brunauer–Emmett–Teller (BET) equation on the N_2_ adsorption isotherms.

*^b^* by the t-plot method based on the adsorption isotherms.

Table S3 Carbon balance of FER zeolites at 350 ^o^C.

| Samples | 0.17 h | 1.17 h | 2.17 h | 3.17 h |
| --- | --- | --- | --- | --- |
| H-FER(Diox) | 96% | 99% | 98% | 96% |
| H-FER(Pyrr) | 96% | 96% | 96% | 96% |
| H-FER(Tosoh) | 100% | 100% | 100% | 100% |

*^a^ Carbon balance* =$\frac{\sum(i*Ci)+CH4}{initial CH4}$

**Reference**

[1] Z. Xiong, G. Qi, L. Bai, E. Zhan, Y. Chu, J. Xu, N. Ta, A. Hao, F. Deng, W. Shen,*Catal. Sci. Technol.* **2022**, *12*, 4993-4997.

[2] A. B. Pinar, P. A. Wright, L. Gómez-Hortigüela, J. Pérez-Pariente, *Micropor Mesopor Mat* **2010**, *129*, 164-172.

[3] G. Kresse, J. Furthmuller, Phys. Rev. B: Condens. Matter Mater. Phys. **1996**, 54, 11169−11186.

[4] G. Kresse, D. Joubert, Phys. Rev. B: Condens. Matter Mater. Phys. **1999**, 59, 1758−1775.

[5] J. P. Perdew, K. Burke, M. Ernzerhof, Phys. Rev. Lett. **1996**, 77, 3865-3868.

[6] T. Lu, F. Chen, J. Comput. Chem. **2012**, 33, 580-592.

[7] W. Humphrey, A. Dalke, K. Schulten, J. Molec. Graphics **1996**, 14.1, 33-38.
